# Association of specific *ACE2* and *TMPRSS2* variants with circulatory cytokines of COVID-19 Emirati patients

**DOI:** 10.3389/fimmu.2024.1348229

**Published:** 2024-05-24

**Authors:** Noha M. Elemam, Amal Bouzid, Habiba Alsafar, Samrein BM Ahmed, Shirin Hafezi, Thenmozhi Venkatachalam, Leen Eldohaji, Tasneem Al Hamidi, Peter Habib Gerges, Nour Halabi, Hassen Hadj-Kacem, Iman M. Talaat, Jalal Taneera, Nabil Sulaiman, Azzam A. Maghazachi, Qutayba Hamid, Rifat Hamoudi, Maha Saber-Ayad

**Affiliations:** ^1^ Research Institute for Medical and Health Sciences, University of Sharjah, Sharjah, United Arab Emirates; ^2^ Department of Clinical Sciences, College of Medicine, University of Sharjah, Sharjah, United Arab Emirates; ^3^ Center for Biotechnology, Khalifa University of Science and Technology, Abu Dhabi, United Arab Emirates; ^4^ Department of Biomedical Engineering, College of Engineering, Khalifa University of Science and Technology, Abu Dhabi, United Arab Emirates; ^5^ Emirates Bio-Research Centre, Ministry of Interior, Abu Dhabi, United Arab Emirates; ^6^ College of Health, Wellbeing and Life Sciences, Department of Biosciences and Chemistry, Sheffield Hallam University, Sheffield, United Kingdom; ^7^ Department of Physiology and Immunology College of Medicine and Health Sciences, Khalifa University of Science and Technology, Abu Dhabi, United Arab Emirates; ^8^ School of Information Technology and Computer Science (ITCS), Nile University, Giza, Egypt; ^9^ Al Jalila Genomics Center of Excellence, Al Jalila Children’s Specialty Hospital, Dubai, United Arab Emirates; ^10^ Department of Applied Biology, College of Sciences, University of Sharjah, Sharjah, United Arab Emirates; ^11^ Pathology Department, Faculty of Medicine, Alexandria University, Alexandria, Egypt; ^12^ Department of Basic Medical Sciences, College of Medicine, University of Sharjah, Sharjah, United Arab Emirates; ^13^ Department of Family Medicine, College of Medicine, University of Sharjah, Sharjah, United Arab Emirates; ^14^ Meakins-Christie Laboratories, Research Institute of the McGill University Health Center, Montreal, QC, Canada; ^15^ Division of Surgery and Interventional Science, University College London, London, United Kingdom; ^16^ College of Medicine, Cairo University, Giza, Egypt

**Keywords:** coronavirus, gene association, population genetics, cytokine, prognostic model, site-direct mutagenesis

## Abstract

**Introduction:**

The COVID-19 pandemic represented one of the most significant challenges to researchers and healthcare providers. Several factors determine the disease severity, whereas none alone can explain the tremendous variability. The Single nucleotide variants (SNVs) in angiotensin-converting enzyme-2 *(ACE2)* and transmembrane serine protease type-2 (*TMPRSS2)* genes affect the virus entry and are considered possible risk factors for COVID-19.

**Methods:**

We compiled a panel of gene variants from both genes and used *in-silico* analysis to predict their significance. We performed biological validation to assess their capacity to alter the *ACE2* interaction with the virus spike protein. Subsequently, we conducted a retrospective comparative genome analysis on those variants in the Emirati patients with different disease severity (total of 96) along with 69 healthy control subjects.

**Results:**

Our results showed that the Emirati population lacks the variants that were previously reported as associated with disease severity, whereas a new variant in *ACE2* “Chr X:g.15584534” was associated with disease severity specifically among female patients. *In-silico* analysis revealed that the new variant can determine the *ACE2* gene transcription. Several cytokines (GM-CSF and IL-6) and chemokines (MCP-1/CCL2, IL-8/CXCL8, and IP-10/CXCL10) were markedly increased in COVID-19 patients with a significant correlation with disease severity. The newly reported genetic variant of *ACE2* showed a positive correlation with CD40L, IL-1β, IL-2, IL-15, and IL-17A in COVID-19 patients.

**Conclusion:**

Whereas COVID-19 represents now a past pandemic, our study underscores the importance of genetic factors specific to a population, which can influence both the susceptibility to viral infections and the level of severity; subsequently expected required preparedness in different areas of the world.

## Introduction

The COVID-19 pandemic represented a cardinal unprecedented challenge to the healthcare systems, economy, and global policy-making. Severe cases of COVID-19 required intensive care unit (ICU) admission and showed increased plasma levels of key inflammatory cytokines leading to cytokine release syndrome (cytokine storm) that correlated with disease severity and mortality due to severe acute respiratory syndrome coronavirus 2 (SARS-CoV-2) virus. A few studies suggested potential prognostic parameters of the disease (e.g., cytokines, D-dimer, C reactive protein, etc.). In our previous study, we identified the potential of certain cytokines such as IL-10 and TNF-α to predict the severity and clinical outcome of COVID-19, using mathematical modeling (1).

Despite the advances in vaccine development, COVID-19 persisted as a worldwide pandemic, characterized by patients displaying a range of symptoms and degrees of severity. Multiple factors were reported to affect the severity and complications, such as age, gender, comorbidities, and geographical variation (2). The latter could be associated with gene variation (mostly single nucleotide variants-SNVs) of the host receptors responsible for virus entry. Recently, Augusto et al. revealed a protective role of HLA-B15:01 allele (3). Previous genome-wide association studies, including one in the UAE, suggested several gene variants associated with the severity of the disease and hospitalization of COVID-19 patients (4, 5). In the meta-analysis by Pairo-Castineira et al., potentially druggable targets were identified including *TMPRSS2* among several genes covering a spectrum of biological systems (6).

The SARS-CoV-2 enters human cells by binding to ACE2 or TMPRSS2; cell surface proteins that facilitate virus entry into the host cell (7). Primarily, ACE2 is known to play a role in maintaining blood pressure, and interestingly, a critical role in the occurrence of acute lung injury in COVID-19. Among the reasons of SARS-CoV-2 being highly infectious is its high binding affinity to ACE2 compared to the previous SARS-CoV strains. Natural sequence variation in human ACE2 protein due to different codes in an individual’s DNA can allow the virus to bind to ACE2 with variable affinities and alter the disease’s susceptibility and severity (8). Researchers still attempt to link such parameters with the genetic aspects of the patients to explain the disease outcome and possible required treatment. SARS CoV-2 spike protein (S protein) allows the binding of the virus to the receptor, hence its internalization to the inside of the mammalian cells. Before binding, the S protein is modified by mammalian membrane serine proteases such as TMPRSS2 (7).

In this study, we aimed to investigate the potential correlation between genetic variants related to viral entry (*ACE2* and *TMPRSS2*) and the presence of circulating cytokines in COVID-19 patients within the United Arab Emirates (UAE) population. By doing so, we aimed to shed additional light on how these host factors contribute to the severity and advancement of COVID-19 infection.

## Subjects, materials, and methods

### Cell lines, constructs, and antibodies

Human lung cancer cell line A549 was obtained from the European Collection of Authenticated Cell Cultures (Salisbury, UK) and maintained in RPMI-1640 medium (Sigma Aldrich, UK) supplemented with 10% fetal bovine serum (FBS) and 1% penicillin/streptomycin (All from Sigma Aldrich, UK). The cells were incubated at 37°C humidified sterile incubator with 5% CO_2_.

The constructs: ACE2 (NM_021804.2) EX-U1285-M35 (c-FLAG), Spike: EX-CoV219-M03 (eGFP at C and CMV), and control vector: EX-EGFP-M03, were obtained from Genecopoeia, USA. The following antibodies were obtained for western blotting: anti-FLAG antibody (Cat no. F1084, Sigma Aldrich, UK), anti-GFP antibody (Cat no. sc-9996, Santa Cruz, USA), and anti-Beta actin (Cat no. A5441, Sigma Aldrich, UK).

### Site-directed mutagenesis

Variants were introduced into the *ACE2* sequence using QuikChange^®^ Site-Directed Mutagenesis Kit (Cat no. 200522, Stratagene, USA). The mutagenesis reaction was set by adding 50 ng of the double-stranded template, 125 ng of both mutated forward and reverse primers-containing desired variants, 1x reaction buffer, 1.25 mM dNTPs,1 µl (2.5 U/µl) Pfu polymerase, and completed till 50 µl using sterile nuclease-free dH_2_O. The reaction was incubated as per the manufacturer’s instructions. The elongation time was set for 11:00 min and 16 cycles.

The methylated template DNA was digested using 1 µl of the Dpn1 endonuclease for 1 hr at 37°C. The mutagenesis product was transformed into DH5α competent bacteria according to the protocol supplied with the kit. A mini-culture was set up from one colony of the transformed bacteria, and plasmid DNA was extracted using Wizard^®^ Plus SV Minipreps DNA Purification System (A1330, Promega, USA). The presence of the introduced variant was confirmed by Sanger sequencing.

### Co-immunoprecipitation and western blotting

A549 cells were cultured to reach 80% confluency. Then, the cells were co-transfected with GFP+FLAG-ACE2, GFP-S+FLAG-ACE2 wild type (Wt), or GFP-S+FLAG-mutated *ACE2* using ViaFect transfection reagent (Promega, USA), according to the manufacturer’s guidelines. After 24 hours, the cells were lysed and 10 μg of proteins were resolved to represent the whole cell lysates, while the rest of the lysates were incubated with immobilized anti-FLAG antibody. The lysates and the beads were incubated overnight at 4°C, then washed and the coimmunoprecipitated proteins were separated from the beads by adding 1X Laemmli buffer sample buffer. The proteins were resolved on SDS-PAGE gel. Immunoblotting was performed using an anti-FLAG antibody, anti-GFP antibody, or anti-beta actin. Band intensity was measured using ImageJ-Win64 software (9).

### Recruitment of healthy controls and COVID-19 patients

In total, 96 COVID-19 patients and 69 healthy control Emirati nationals were recruited in this study. The COVID-19 patients (mild, moderate and severe) were collected from several hospitals in Dubai and Abu Dhabi, UAE (DSREC-04/2020_09 on April 26, 2020). No exclusion criteria was set regarding gender or co-morbidities. The healthy controls were taken prior to the first instance of COVID-19 infection in the UAE (MO-HAP/DXB/SUBC/No.14/2017). Heathy controls were filtered to include only those with a non-obese BMI and normal HbA1c to avoid having any confounding factors such as obesity or prediabetes. All recruited patients and controls were above 18 years old. Blood samples were collected, after which it was processed for serum collection and DNA extraction. The demographic and clinical characteristics of the recruited COVID-19 patients and healthy controls are summarized in **Table 1**.

**Table 1:** Demographic and clinical characteristics of the recruited COVID-19 patients and healthy controls.

	Healthy controls (n=69)
**Gender (Males/Females)**	27/42
**Age (Mean ± SD)**	40.2 ± 15.7
**BMI (Mean ± SD)**	25.4 ± 2.90
	COVID-19 patients (n=96)
Severity	
Mild	32
Moderate	32
Severe	32
**Gender (Males/Females)**	74/22
**Age (Mean ± SD)**	51.5 ± 19.7
**BMI (Mean ± SD)**	32 ± 20.95
Symptoms	Number of patients (n, %)
Respiratory symptoms	92, 95.8
Constitutional symptoms	83, 86.5
Loss of Taste or Smell	32, 33.3
GIT symptoms	39, 40.6
Conjunctivitis	2, 2.1
Management	
Hospitalization	73, 76
Oxygen Therapy Required	57, 59,4
ICU Admission	50, 52.1
Mechanical Incubation	21, 21.9
Comorbidities	
Cardiac Disease	10, 10.4
Chronic Lung Disease	9, 9.4
Haemoglobiopathies	0, 0
Type 2 Diabetes	42, 43.8
Immunosuppressive Condition	7, 7.3
Liver Disease	5, 5.2
Metabolic Disease	29, 30.2
Neurological Disorder	5, 5.2
Renal Disease	23, 24
**Smoking**	12, 12.5
COVID-19 Vaccine	
None	15, 15.6
1st dose	6, 6.25
2nd dose	12, 12.5
Unknown	60, 62.5
Therapies	
Antibiotic	3, 3.18
Antiviral	68, 70.8
Non specific immunomodulator	59, 61.4
Tocilizumab	5, 5.2
Anticoagulant	33, 34.4
Vitamin C	0, 0
Stem cell therapy	2, 2.1

### Serum collection and DNA extraction

Whole blood samples were centrifuged, and serum was collected and frozen at -20°C for cytokine analysis at a later point. The blood samples were preserved at −20°C to perform DNA extraction using a QIAamp extraction kit (Cat no. 51306, Qiagen, Germany).

### Targeted amplicon sequencing

Primer sets were designed to cover the exonic and intronic regions of the *ACE2* and *TMPRSS2* genes. The details of the primers are listed in **Supplementary Table S1**. The targeted amplicon size was about 200 bp. First, all primers were assessed using control DNA samples, and thus, the expected amplicon sizes were validated using conventional PCR. Then, the library for targeted next-generation sequencing was prepared using the Fluidigm 48.48 Access Array integrated fluidic circuit (IFC) (Fluidigm Europe B.V, Netherlands) as previously described (10). The purified amplicon libraries were diluted to 1 pg and then amplified using emulsion PCR with Ion Template OT2 kit in Ion OneTouch™ ES system following the manufacturer’s instructions (ThermoFisher, USA). Next, all samples of COVID-19 patients and healthy controls were equally pooled and sequenced using the Ion 520™ Chip on an Ion S5 XL Semiconductor (ThermoFisher, USA).

### Genomic data processing and variant identification

The genomic data were processed using an in-house bioinformatics pipeline including alignment to the reference genome, quality control evaluation, variant calling and variant annotation as previously described (11). Coverage analysis was applied to the flanking sequences at the gene level of the target regions. To further warranty the accuracy of variant and genotype calling, variants with low coverage < 10 and base quality score < 10 were excluded from the downstream variant analysis. Functional annotation of the variants was performed using the Ensembl Variant Effect Predictor tool (12). Variant frequency evaluation among different populations was carried out using large genomic databases including Sequence (RefSeq), Single Nucleotide Polymorphism database (dbSNP), 1000 Genomes, and Exome Aggregation Consortium (ExAC). Exonic variants were further assessed for pathogenicity using PolyPhen2 and SIFT tools (13, 14).

### Hierarchical clustering and linkage disequilibrium analysis

Unsupervised hierarchical clustering including Euclidean and Ward’s linkage method was applied using an in-house R script to assess the possible grouping distribution of the identified variants within the *ACE2* and the *TMPRSS2* genes based on the minor allele frequency across different populations. This multivariate analysis calculates a square matrix of pairwise distances between the SNVs to be clustered and thus SNVs with similar genetic patterns will be grouped under the same cluster. In addition, in order to assess the linkage disequilibrium (LD) between the identified SNVs in each gene *ACE2* and *TMPRSS2*, pairwise LD was assessed among the general population. LD data were obtained from LDlink (15). SNVs in (LD) exhibit a correlation, with the degree of correlation typically increasing as the variants are closer in physical distance. The LD was estimated by the metrics (D’), which reflects the difference between the observed and the expected frequency of a given haplotype, and (R^2^) which presents the correlation between a pair of loci. D’ values vary between 0 and 1 with higher values indicating tight linkage of alleles and R^2^ values vary between 0 and 1 with higher values indicating a higher degree of correlation.

### *In silico* analyses

Several *in silico* analyses were performed to predict the roles and regulatory effects of non-coding variants. The Regulome DB database (16) was used to explore the DNA features and regulatory elements and the HaploRegdatabase v4.1 (17) was searched to predict the impact of SNVs on regulatory motifs.

### Cytokine assay

A 50 μl of each of the serum samples of COVID-19 patients and healthy controls were used for the assessment of cytokines that were previously analyzed by our group in COVID-19 patients (1). The Human Immunotherapy Magnetic Luminex Performance Assay 24-plex Fixed Panel (R&D systems, USA) and the Bioplex-200 system (Bio-Rad Laboratories, USA) were used.

### Statistical analysis

Descriptive characteristics were presented as means and standard deviations or as frequencies and percentages. One-way analysis of Variance (ANOVA), followed by Bonferroni correction as a *Post Hoc* Test to control the familywise error rate, was used to assess differences in variant severity across the distinct COVID-19 patient groups: mild, moderate, and severe. The Chi-square test was applied to compare the variant frequencies between the COVID-19 patients and healthy controls. Multivariate logistic regression analysis was carried out to assess associations between variants and gender. Analysis of cytokine levels between the two groups was done using the Mann-Whitney U test. Pearson correlations linear regression was performed to evaluate correlations between the identified variants in *ACE2* and *TMPRSS2* genes and the different inflammatory cytokines levels among COVID-19 patients and healthy controls. Statistical analyses were performed using Graph Pad Prism 6 (Graph Pad, San Diego, CA, USA) and IBM SPSS Statistics software version 23 (IBM Corp, Armonk, NY, USA). Error bars represent the standard error of the mean (SEM), and two-tailed p-value (p) < 0.05 was considered statistically significant for all statistical tests.

## Results

### Key variants in the *ACE2* receptor

Based on several previous studies, 15 non-synonymous variants with high frequency in Asians (14 SNVs and 1 INDEL) for ACE2 were selected (**Table 2**) (18–28). Most of the 15 variants had higher allele frequency (AF) in the ChinaMAP dataset and EAS populations compared to European populations [10]. Polyphen-2 tool was utilized for its ability to predict the effect of a variant on the resultant protein for the selected 15 variants [11]. Six variants appeared to have a potentially damaging effect on ACE2 protein: S19P, G211R, P263S, S692P, A501T, and L731F. Furthermore, TMPRSS2 is a protease that mediates the processing of the S protein and supports viral entry. Following the same approach, we also included TMPRSS2 variants. As targeted sequencing was planned, primers were designed to cover more variants (all previously reported SNVs of known significance in the ACE2 and TMPRSS2 genes, including splice regions).

**Table 2:** Characteristics of ACE2 selected variants.

Allele	Uploaded Allele	Protein position	Amino acid	Codons	Consequence	Feature	Biotype	MANE SELECT	SIFT	PolyPhen	Functional consequences	References
C	T/C	720	N/D	Aac/Gac	missense_variant	ENST00000252519.8	protein_coding	NM_001371415.1	tolerated_low_confidence(0.28)	benign(0.021)	Predicted to interfere with protein structure and stabilization	(18)
G	A/G	692	S/P	Tct/Cct	missense_variant	ENST00000252519.8	protein_coding	NM_001371415.1	deleterious_low_confidence(0.02)	possibly_damaging(0.731)	Predicted to strongly influence the function and stability of ACE2	(19)
G	A/G	19	S/P	Tcc/Ccc	missense_variant	ENST00000252519.8	protein_coding	NM_001371415.1	tolerated(0.09)	possibly_damaging(0.748)	Showed noticeable variations in their intermolecular interactions with the viral spike protein	(20)
C	T/C	468	I/V	Att/Gtt	missense_variant	ENST00000252519.8	protein_coding	NM_001371415.1	deleterious(0.04)	possibly_damaging(0.661)	Molecular dynamic demonstrated that two of these variants (K26R and I468V) may affect binding characteristics between S protein of the virus and hACE2 receptor	(21)
C	T/C	341	K/R	aAa/aGa	missense_variant	ENST00000252519.8	protein_coding	NM_001371415.1	deleterious(0.04)	benign(0.154)	affecting stability in the ACE2 - SARS-CoV-2-S complex.	(22)
A	C/A/T	614	A/S	Gca/Tca	missense_variant,splice_region_variant	ENST00000252519.8	protein_coding	NM_001371415.1	tolerated (1)	benign(0)	benign	(23)
T	C/T	211	G/R	Ggg/Agg	missense_variant	ENST00000252519.8	protein_coding	NM_001371415.1	tolerated(0.32)	benign(0.351)	may affect secondary structures	(24)
T	C/T	582	R/K	aGg/aAg	missense_variant	ENST00000252519.8	protein_coding	NM_001371415.1	tolerated(1)	benign(0.051)	benign	(23)
T	C/T	501	A/T	Gca/Aca	missense_variant	ENST00000252519.8	protein_coding	NM_001371415.1	tolerated(0.06)	benign(0.127)	alters affinity	(25)
C	A/C	656	L/*	tTa/tGa	stop_gained	ENST00000252519.8	protein_coding	NM_001371415.1	–	–	reduced protein function	(26)
G	A	731	L/F	Ctt/Ttt	missense_variant	ENST00000252519.9	protein_coding	NM_001371415.2	Moderate	probably_damaging(0.943)	predicted to strongly influence the function and stability of ACE2.	(19)
G	A	263	P/S	Cct/Tct	missense_variant	ENST00000252519.10	protein_coding	NM_001371415.3	deleterious (0)	probably_damaging(1)	significantly reduces ACE2 protein expression levels	(27)
T	C	26	K/R	aAg/aGg	missense_variant	ENST00000252519.11	protein_coding	NM_001371415.4	tolerated(0.84)	benign(0)	affecting stability in the ACE2 - SARS-CoV-2-S complex.	(22)
T	C	329	E/G	gAa/gGa	missense_variant	ENST00000252519.12	protein_coding	NM_001371415.5	deleterious(0.03)	benign(0.098)	showed noticeable variations in their intermolecular interactions with the viral spike protein	(20)
T	C	206	D/G	gAc/gGc	missense_variant	ENST00000252519.13	protein_coding	NM_001371415.6	deleterious(0.05)	benign(0.068)	increases the electrostatic attraction	(28)

### Developing the adopted system

**Figure 1:**
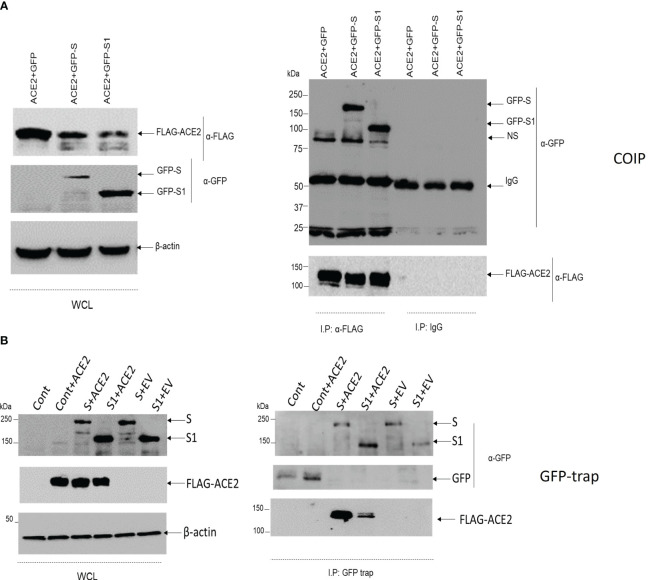
The association of ACE2 with S/S1 using coimmunoprecipitation and GFP trap. **(A)** A549 cells were co-transfected with GFP+ACE2, GFP-S+ACE2 or GFP-S1+ACE2. After 24 hrs, the cells were lysed and 10 ug of the proteins were kept for whole cell lysates (WCL). The rest of the lysates were incubated overnight with Sepharose beads coupled with 2ug of anti-FLAG antibody. The immunoprecipitated proteins were suspended in 2x Laemmli buffer. The protein was resolved on 8% SDS-PAGE gel. Immunoblotting was then performed with anti-FLAG and anti-GFP antibodies. Anti-β actin was used as a loading control antibody. **(B)** A549 cells were either transfected with control GFP vector (cont, Cont+ACE2, S+ACE2, S1+ACE2, S+FLAG tag empty vector (EV), or S1+EV. After 24 hrs, the cells were lysed and 10 mu of the lysates were reserved to represent the transfection in the WCL, while the rest of the lysates were incubated with ChromoTek-GFP-Trap. The precipitated proteins were resolved on 8%SDS-PAGE gel. Immunoblotting was then performed with anti-FLAG and anti-GFP antibodies. Anti-β actin was used as a loading control antibody.

Before further testing the potential damaging variants, it was thought first to check for the validity of the used *in vitro* model. A549 was selected as a testing model since it is originally an alveolar basal epithelial cell line (29). Second, it was crucial to examine whether the GFP-tagged viral S protein interacts with FLAG-tagged ACE2 in the A549 cell line. Co-transfection, as well as coimmunoprecipitation techniques, were employed. The cells were co-transfected with S or S1, a fraction of the S protein, and FLAG-tagged wild-type ACE2. The cells were lysed, and the ACE2-FLAG was immunoprecipitated with an immobilized anti-FLAG antibody (**Figure 1**). FLAG-ACE2 was able to pull down the GFP-tagged S or S1. A GFP trap was also performed to validate this finding (**Figure 1**) and to ensure that the GFP tag does not cause the observed association between ACE2 and S/S1. Due to discrepancies in the pulldowns concerning S1 interactions with ACE2, we chose to explore downstream pulldown testing using GFP-S. Colocalization studies have confirmed the association of FLAG-tagged ACE2 and GFP-tagged S protein in A549 cells. Therefore, this proved that the system is working and full-length wild-type ACE2 does interact with the viral spike protein.

### Testing the interaction of the mutated ACE2 with spike protein

**Figure 2:**
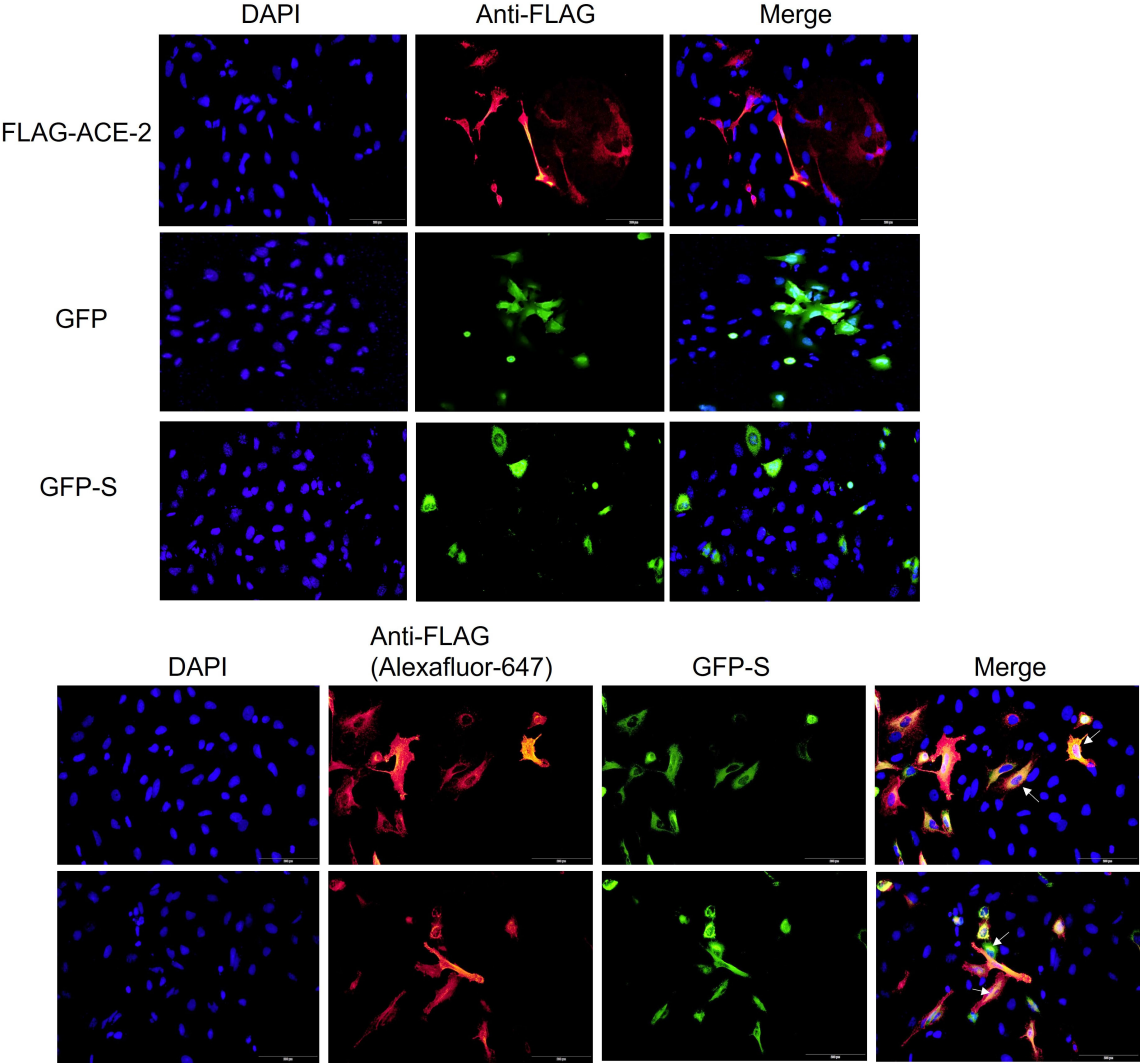
ACE2 and Spike colocalization in A549 cells. The cells were transfected with FLAG-ACE2, GFP, or GFP-Spike (S) or co-transfected with the FLA-ACE2 and GFP-S for 24 hrs using Viafect transfection reagent. After fixing and permeabilizing the cells, FLAG-ACE2 was detected by using an anti-FLAG antibody and secondary antibody coupled with Alex-fluor 647 (red). The cells were mounted using mounting medium with DAPI (blue). The images were acquired by Loympus CellSense software. The scale bar is 100um. Arrows indicate the colocalization of FLAg-ACE2 and GFP-S.

**Figure 3:**
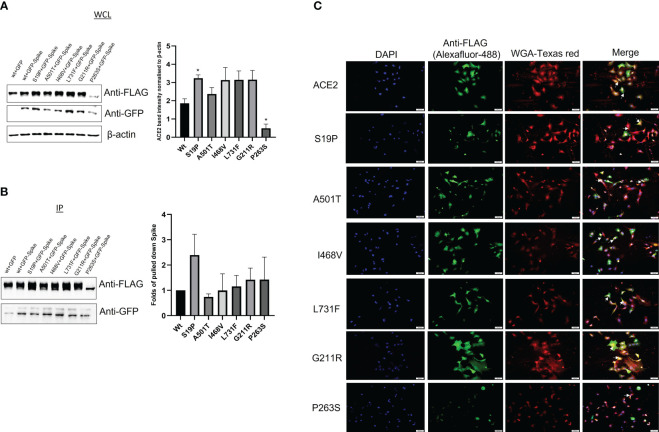
The effect of the ACE-2 varianton the binding to Spike protein. A549 cells were co transfected with either ACE2 (wt)+GFP, ACE2-(Wt)+GFP-S, or mutant ACE2 (S19P, A501T, I468V, L731F, G211R and P263S)+GFP-S. The cell lysates were used for **(A)** whole cell lysate (WCL) or **(B)** immunoprecipitation. Immunoblotting was then performed using anti-FLAG and anti-GFP antibodies. Anti-β actin was used as a loading control antibody. (p<0.05; n=4). ANOVA: p-value=0.0285. **(C)** ACE2 mutants subcellular distribution. A549 cells were seeded on coverslips for 24 hrs. The cells were then transfected with ACE2 wild type (Wt) and its variantss for 24hrs. The cells were stained with a membrane stain, Wheat Germ Agglutinin (WGA), conjugated with Texas red. This followed by fixing and permeabilizing the cells. After the cells were saturated, ACE2 and its mutants were detected by anti-FLAG antibody and Alexafluor-488 secondary antibody. The cells were visualized by Cellsens software. Scale bars; 100 um. Arrow indicates ACE2 membrane localization. * p<0.05.

To investigate the effect of the *ACE2* variants on the binding to viral S protein, site-directed mutagenesis, co-transfection, and coimmunoprecipitation methods were performed. A549 cells were co-transfected with ACE2+GFP, ACE2 (Wt)+GFP-S, or with mutated ACE2+GFP-S (**Figure 2**). Then, the cells were lysed and the expression of the mutated ACE2 was compared to the wild type of *ACE2*. As shown in **Figure 3A**, the protein expression of the mutated ACE-2 (S19P, A501T, I468V, L731F, G211R, and P263S) in A549 cells is higher than the wild-type ACE2 except for the S263P variant that showed significantly reduced expressions (Kruskal–Wallis test, p<0.05; n=4).

An anti-FLAG antibody successfully pulled down *ACE2*, while the ability of the mutated *ACE2* to pull down the S protein was unevenly affected by variant introduction. S19P has positively impacted the interaction between *ACE2* and S protein while the rest of the variants have relatively debilitated the interaction between the ACE2 and the S protein (**Figure 3B**).

### Subcellular localization of the wild-type ACE2 and its mutants in A549 cells

*ACE2* membrane localization is crucial for its binding to spike protein. Therefore, it was decided to investigate whether the ACE-2-associated SNVs affect ACE-2 membrane localization. A549 cells expressing FLAG-ACE2 (Wt) and its mutants were stained with membrane stain, Wheat Germ Agglutinin (WGA). ACE2 was detected with an anti-FLAG antibody and Alexa-fluor-488 antibody. ACE2 wild type and its mutants showed membrane localization (**Figure 3C**). However, interestingly, P263S transfected cells showed less green signal when compared to ACE2 (Wt) and other mutants, which made observing the membrane localization of ACE2 less witnessed. This observation is consistent with less expression noted in **Figure 3A**.

### Targeted genomic amplicon sequencing identified single nucleotide variants in ACE2 and TMPRSS2 among the Emirati population

Based on the previously curated ACE2 and TMPRSS2 SNVs (26, 30, 31), we explored those specific regions on the genome using targeted sequencing. We examined the regions up and downstream the previously reported *ACE2* and *TMPRSS2* SNVs, by generating 150-200-bp amplicons (33 for *ACE2* and 21 for *TMPRSS2*). The targeted genomic amplicon sequencing using the Fluidigm assay was performed on 96 COVID-19 patients and 69 healthy controls. The variants analysis identified 7 single nucleotide variants (SNVs) in *ACE2* and *TMPRSS2*. As shown in **Supplementary Table S2**, for the ACE2 gene, all variants are located in the intronic regions, while, for the TMPRSS2 gene, two variants are intronic and five variants are exonic of which 4 are synonymous variants and one is a missense variant predicted as deleterious and probably damaging by SIFT and PolyPhen, respectively. The prevalence of the identified SNVs in *ACE2* and *TMPRSS2* across all case subgroups is described in **Supplementary Table S3**.

### Association of *TMPRSS2* variants rs28524972 and rs17854725 in men with COVID-19 infection

**Figure 4:**
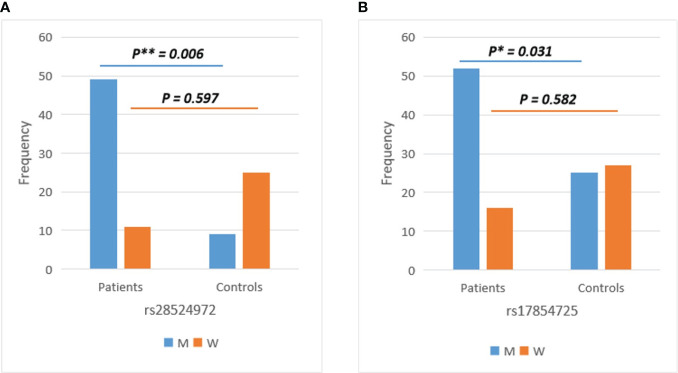
Gender-stratified analysis of *TMPRSS2* variants showed significant differences between COVID-19 patients and healthy controls in men for **(A)** rs28524972 and **(B)** rs17854725. M, men; W, women.

The association of *ACE2* and *TMPRSS2* SNVs with COVID-19 infection was performed among the COVID-19 patients and healthy controls. Case-control association analysis showed no statistically significant differences (p > 0.05, Chi-Square Test) between COVID-19 patients and healthy controls for all identified variants within the *ACE2* and *TMPRSS2* genes. However, the gender-stratified analysis revealed a significant association with COVID-19 infection of *TMPRSS2* SNVs rs28524972 (p=0.006, Chi-Square Test) and rs17854725 (p=0.031, Chi-Square Test) in men susceptibility only (**Figure 4**), while no gender effect was detected for the remaining *TMPRSS2* variants and all SNVs in the ACE2 gene. The association evaluation of *ACE2* and *TMPRSS2* SNVs with COVID-19 infection among the COVID-19 patients and healthy control subjects of the total and gender-stratified cases are listed in **Supplementary Table S4**.

### Potential associations of *ACE2* variant X:g.15584534 with COVID-19 infection severity in the Emirati population

To evaluate the potential association of the identified SNVs with the severity in COVID-19 patients, we evaluated the association between different SNVs and mild, moderate, and severe COVID-19 patients’ subgroups. Notably, the SNV X:g.15584534 showed a significant difference between the three groups (p=0.001, ANOVA test). This novel-identified variant present with an average coverage of about 497 among all subjects showed lower prevalence in mild (81.3%) than in moderate (100%) and severe (100%) subgroups, suggesting this SNV is a marker of severity in COVID-19 patients. The details of the coverage of the *ACE2* X:g.15584534 SNV are presented in **Supplementary Table S5**. In addition, a female gender association with the COVID-19 infection severity was significantly detected in *ACE2* for X:g.15584534 SNV with p=0.02, ANOVA test), respectively. The multiple comparisons with Bonferroni correction of the identified *ACE2* and *TMPRESS2* variants among the COVID-19 patients are summarized in **Supplementary Table S6** and **Supplementary Table S7**, respectively.

### The novel variant X:g.15584534 may affect the transcription of *ACE2*

The *in-silico* function prediction of the non-coding variant X:g.15584534G>A in the intronic region (intron 15, NM_001371415.1) of the *ACE2* gene showed that the genotype G/A has a RegulomeDB score about 0.57, suggesting its probable roles as a transcription factor and/or DNAse peak. Moreover, further functional prediction using UCSC Genome Browser on Human (GRCh37/hg19) revealed this particular variant coincides with several transcription binding sites including *ZNF675, ZNF418, ETV2*, and *DRGX.* Additionally, the variant position was predicted to be conserved (score=0.53) using the Multiz Alignments of 100 Vertebrates. Taken together, we assume that this novel variant X:g.15584534G>A may play an important role in the transcription regulation of the *ACE2* gene, which may consequently affect the susceptibility and severity of COVID-19 infection.

### Profiling of tested SNVs in the Emirati population compared to different ethnicities

The unsupervised hierarchical clustering analysis was performed between the Emirati and other ethnic populations including African, American, East Asian, European, and South Asian based on the minor allele frequency on all identified SNVs in *ACE2* and *TMPRSS2* genes using available data from the 1000Genomes project Phase 3 Continental populations (GRCh37/hg19). The clustering differentiated between the populations and showed that the Emirati population has unique allele enrichment/depletion profiles compared to the other populations suggesting its unique genetic pattern. Particularly, the clustering analysis revealed that the East Asian population is closer to the Emirati population (**Supplementary Figure S1**).

### Linkage disequilibrium of *ACE2* and *TMPRSS2* variants in the general population

The linkage disequilibrium analysis was performed using allele frequency of the general population from publicly available reference haplotypes from the 1000 Genomes Project to evaluate the association between the identified variants in *ACE2* and *TMPRSS2* genes. Importantly, rs879922 and rs4240157 SNVs in the *ACE2* gene showed a high association (D’= 0.989, R^2^ = 0.977) (**Supplementary Figure S2a**) while in *TMPRSS2*, rs12329760, and rs2298659 showed the highest association (D’= 0.735, R^2^ = 0.405) among other SNVs (**Supplementary Figure S2b**).

### Cytokine levels in COVID-19 patients with various severities

It was our interest to explore the levels of 24 cytokines previously linked to the prediction and severity of COVID-19 infection (1) in healthy controls and COVID-19 patients (**Supplementary Table S8**).

**Figure 5:**
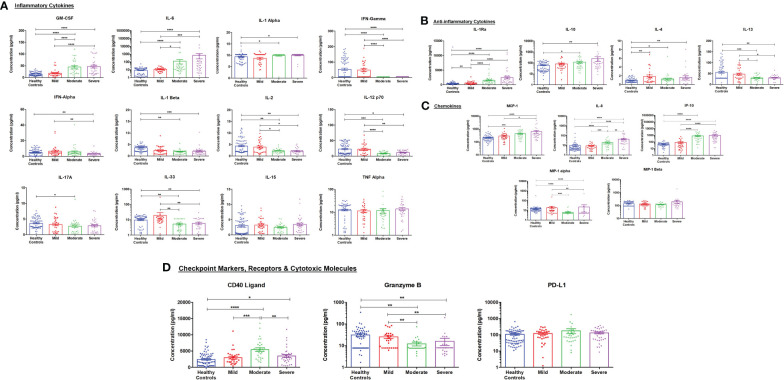
Cytokine assessment in healthy control subjects (n =69), mild (n=29), moderate (n=) COVID-19 (n= 31) and severe (n=32) COVID-19 patients. **(A)** Inflammatory, **(B)** anti-inflammatory cytokines, **(C)** chemokines, and **(D)** checkpoint markers, receptors, and cytotoxic mediators were assessed in mild, moderate and severe COVID-19 patients and their levels compared to healthy controls. Data is expressed as mean ± standard error of the mean (SEM). *p<0.05, **p<0.01, ***p<0.001, and ****p<0.0001.

COVID-19 infection was associated with cytokine storm, where several cytokines were measured in mild, moderate, and severe COVID-19 patients compared to healthy controls. As shown in **Figure 5A**, several cytokines were elevated in COVID-19 patients with a dramatic increase in disease severity such as GM-CSF and IL-6. IL-1α was found to show a slight elevation in its levels in moderate and severe COVID-19 patients. Interestingly, several cytokines such as IFN-γ, IFN-α, IL-1β, IL-2, IL-12p70, IL-17A and IL-33) decreased in moderate and severe COVID-19 patients compared to mild cases or healthy controls.

Regarding the anti-inflammatory cytokines, IL-1 receptor antagonists (IL-1Ra), IL-10, and IL-4 significantly increased across the different groups (mild, moderate, and severe) of COVID-19 patients. In contrast, IL-13 was found to be reduced in moderate and severe COVID-19 patients compared to mild COVID-19 patients or healthy controls (**Figure 5B**).

Five chemokines were explored in this study, where 3 of them (MCP-1/CCL2, IL-8/CXCL8, and IP-10/CXCL10) showed a significant increase in COVID-19 patients with further increase in higher severities. On the other hand, MIP-1α was reduced in moderate and severe COVID-19 patients compared to healthy controls, while MIP-1β did not show any significance in the levels across the different groups (**Figure 5C**).

Lastly, CD40 ligand (CD40L) was significantly higher in moderate and severe COVID-19 patients. In contrast, the cytotoxic mediator, granzyme B, was found to be reduced in these groups of COVID-19 patients. The checkpoint marker, PD-L1, showed no significant difference across the groups (**Figure 5D**). **Table 3** summarizes the significant associations between the cytokine levels and COVID-19 severity where GM-CSF, IL-1α, IL-1Ra, IL-8, IP-10, and MCP-1 showed significant positive correlation patterns while IL-12p70, IL-13, IL-2, and IFN-γ showed significant negative correlation patterns with COVID-19 severity.

**Table 3:** Association of the cytokine levels and COVID-19 severity.

Cytokines associated with COVID-19 severity	r value	p value
**GM-CSF**	0.371	<0.001
**IL-1α**	0.257	0.015
**IL-1Ra**	0.413	<0.001
**IL-8**	0.421	<0.001
**IP-10**	0.341	0.001
**MCP-1**	0.243	0.021
**IL-12p70**	-0.344	<0.001
**IL-13**	-0.314	0.003
**IL-2**	-0.363	<0.001
**IFN-γ**	-0.529	<0.001

### Correlation between the prevalence of SNVs and cytokine levels

**Figure 6:**
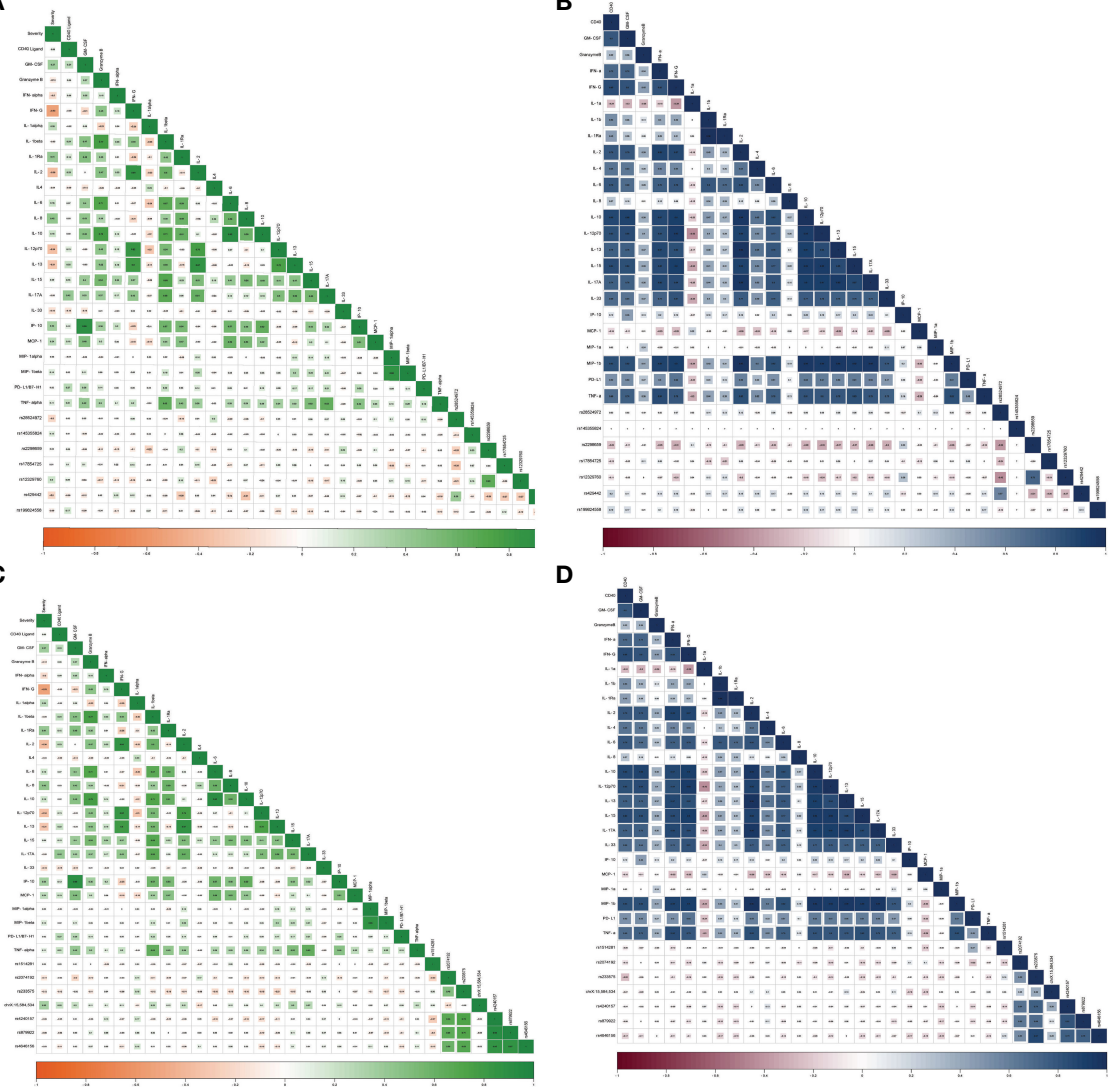
Correlation between inflammatory cytokines and SNVs in *TMPRSS2* among **(A)** COVID-19 patients and **(B)** healthy controls as well as SNVs in *ACE2* among **(C)** COVID-19 patients and **(D)** healthy controls.

Next, it was critical to explore if there is any correlation between the prevalence of the identified SNVs of *ACE2* and *TMPRSS2* with the 24 investigated cytokines in COVID-19 patients (**Figures 6A, B**) and healthy controls (**Figures 6C, D**).

*ACE2* variants showed significant correlations with the investigated cytokines. rs1514281 SNV of the *ACE2* gene showed a significant positive correlation with PD-L1 in healthy controls. Additionally, in healthy controls, rs233575 SNV of the *ACE2* gene showed a negative significant correlation with CD40L, while in COVID-19 patients, the SNV rs233575 of the *ACE2* gene showed a negative significant association with IL-13. Interestingly, the genetic variant chrX:15,584,534_ of the *ACE2* gene correlated positively with CD40L, IL-1β, IL-2, IL-15, and IL-17A in COVID-19 patients (**Table 4**).

**Table 4:** Correlation between inflammatory cytokines and TMPRSS2 and ACE2 SNPs in healthy controls and COVID-19 patients.

SNP	Cytokine	Healthy Controls		COVID-19 Patients	
		r value	p-value	r value	p-value
**rs2298659_TMPRSS2** ** ** ** ** ** ** ** ** ** ** ** ** ** ** ** ** ** **	**IFN-α**	**-0.248**	0.04 *	-0.131	0.22 ^ns^
	**IFN-γ**	**-0.308**	0.01 *	-0.104	0.329 ^ns^
	**IL-10**	**-0.291**	0.014 *	-0.016	0.878 ^ns^
	**IL-2**	**-0.294**	0.015 *	-0.104	0.331 ^ns^
	**IL-12p70**	**-0.328**	0.006 *	-0.063	0.557 ^ns^
	**IL-13**	**-0.267**	0.027 *	-0.065	0.545 ^ns^
	**IL-15**	**-0.273**	0.023 *	-0.153	0.149 ^ns^
	**IL-17A**	**-0.285**	0.018 *	-0.112	0.292 ^ns^
	**IL-33**	**-0.300**	0.012 *	**0.229**	0.03 *
	**MIP-1β**	**-0.263**	0.029 *	-0.066	0.534 ^ns^
	**IL-1α**	0.100	0.412 ^ns^	**-0.228**	0.03 *
**rs12329760_TMPRSS2**	**IP-10**	**0.295**	0.014 *	-0.089	0.405 ^ns^
**rs17854725_TMPRSS2**	**MIP-1α**	0.062	0.611 ^ns^	**-0.219**	0.038 *
**rs429442_TMPRSS2** ** **	**IL-1Ra**	0.17	0.163 ^ns^	**-0.276**	0.008 *
	**IL-8**	-0.018	0.881 ^ns^	**-0.311**	0.003 *
**rs1514281_ACE2**	**PD-L1**	**0.367**	0.002 *	-0.072	0.497 ^ns^
**rs233575_ACE2**	**CD40L**	**-0.245**	0.042 *	-0.115	0.279 ^ns^
	**IL-13**	-0.049	0.689 ^ns^	**-0.212**	0.045 *
**chrX:15,584,534_ACE2**	**CD40L**	-0.069	0.572 ^ns^	**0.216**	0.041 *
	**IL-1β**	0.04	0.744 ^ns^	**0.223**	0.027 *
	**IL-2**	0.098	0.423 ^ns^	**0.213**	0.044 *
	**IL-15**	-0.062	0.613 ^ns^	**0.301**	0.004 *
	**IL-17A**	0.025	0.841 ^ns^	**0.272**	0.01 *

Significant data is shown in bold. ns, not signifcant; * p<0.05.

Regarding the *TMPRSS2* variants, and in healthy controls, the rs2298659 SNV was negatively correlated with multiple cytokines including IFN-α, IFN-γ, IL-10, IL-2, IL-12p70, IL-13, IL-15, IL-17A, IL-33, and MIP-1β. However, in COVID-19 patients, it was negatively correlated with IL-1α and IL-33. Moreover, the rs12329760 SNV showed a positive correlation with IP-10 serum levels in healthy controls. However, such a correlation was not reported in COVID-19 patients. Other *TMPRSS2* variants such as rs17854725 SNV, showed a significant negative correlation with MIP-1α, while rs429442 SNV showed a significant negative correlation with IL-1Ra and IL-8 in COVID-19 patients (**Table 4**).

## Discussion

Substantial genetic diversity avails in the human genome, influencing not only human characteristics but also susceptibility to diseases and their outcomes. One of the current cumbersome diseases that indeed demonstrated the inordinate disparity among different individuals is COVID-19. Variable individuals’ susceptibility, a wide range of severity as well as inconsistent outcomes are features of COVID-19 infection. Several factors were suggested to explain this disparity, such as age, gender, and genetic susceptibility, without definitive conclusions. Here, we aimed to study the interaction of a specific set of *ACE2* SNVs that might either coincide with previous studies or introduce unique insights exclusive to this study. To achieve this, we adopted a comprehensive strategy, i.e., using a bioinformatic tool (Polyphen-2) and wet lab experiments such as co-transfection and coimmunoprecipitation.

The expression of the mutant *ACE2* varied when compared to the wild type of the protein, where S19P showed remarkable increments in *ACE2* expression. In parallel, P263S had negatively affected the *ACE2* expression with no impact on S protein binding. Proline substitution was shown to affect the protein synthesis and stability rate, while minimum evidence was reported for its interference with protein function (32).

Subcellular distribution of ACE2 showed predominant distribution in both cytoplasmic and nuclear compartments. Nuclear localization of ACE2 was found to contribute to SARS-CoV-2 replication (33). Furthermore, ACE2 membrane localization is necessary for SARS-CoV-2 internalization (7); therefore, this study sought to investigate the impact of the introduced variants on the membrane localization of ACE2. None of the *ACE2* variants has fully obliterated ACE2 membrane localization. On the other hand, colocalization studies revealed that ACE2 receptor and SARS-CoV-2 Spike protein association at the perinuclear region. SARS-CoV-2 is usually located in the early endosomes at the perinuclear region (34).

According to the GenomAD database, S19P has 0.03% frequency among different ethnic backgrounds (35). A study by Barton et al. showed that S19P increases the affinity to S Protein by 3.7-folds (36), which is similar to our findings. S19P is frequent among African/African-Americans, albeit the mild state of COVID-19 infection among the African population. This highlights that other factors might have a more influential impact on the severity of the disease such as that observed in the malarial infection (37). A501T is one of the frequent variants in the European population. Previously, studies employing OncoMX, a tool with high biomarker entries, revealed A501T, G211R, and I468V variants where ACE2 has a high affinity to bind to the S protein (25). Inconsistent with this study, our findings showed that G211R had slightly caused an increase; however insignificant, in the binding affinity of ACE2 to the S protein. Furthermore, I468V variant showed no impact on the binding, whereas A501T resulted in a reduced interaction between ACE2 and S protein.

L731 was predicted to affect the ACE2-SLC6A19/B0AT1 association based on FireDock modeling (38). Lung pneumocytes lack the expression of B0AT1, unlike the intestine. For this reason, the metalloproteinases TMPRSS2 and ADAM17 have access to ACE2 cleavage which eases the virus entry and a constrained lung pathology (39). The ACE2/B0AT1 association occurs in the enterocytes; thereby the ACE2 is protected against cleavage by the metalloproteinases, which provide resistance against the virus binding (39). L731 variant was identified to affect this ACE2/B0AT1 interaction [18] negatively. This report shows no noted difference (1.15 folds) in the binding affinity of the L731 mutant compared to the wild type in the lung A549 cells.

In a study by Rokni et al. on a closely related population, different minor alleles of all studied *TMPRSS2* variants statistically increased the risk of COVID-19 (40). Close to our findings of association of this variant with the COVID-19 infection in men, their study concluded that *rs17854725 A > G* (*AA* vs. *AG* and *AA* vs. *GG*), among a few other SNVs of *TMPRSS2* variations, is associated with severe COVID-19. Their statistical analysis revealed that the G allele of *rs17854725* enhanced the risk of COVID-19. In their model, consistent with our findings, the homozygous G vs. homozygous A codominant model was associated with an increased risk of COVID-19. In a study from Mexico by Posadas-Sánchez et al., 2022, the rs2298659 was one of the SNVs associated with a high risk of developing COVID-19, as demonstrated by using different inheritance models (41). Intriguingly, in our current study, it has a negative correlation with several cytokines in healthy control, but with only IL-1α and IL-33 in COVID-19 patients, denoting the variable response to SARS-CoV-2 infection in different ethnicities. TMPRSS2 was previously suggested as a promising drug target for COVID-19 by using camostat mesylate, a drug approved for treating chronic pancreatitis and postoperative reflux esophagitis (42). The gene variant effect on the drug affinity to TMPRSS2 is not determined. The *TMPRSS2* SNVs as mentioned earlier, may be a therapeutic/pharmacogenetic marker of the response to camostat therapy.

Previous reports evaluated the effect of *ACE2* variants on COVID-19 disease risk and severity independently from other risk factors. Senko et al. suggested a few *ACE2* SNVs; however, all were not statistically significant in our cohort (43). In contrast, in our cohort, SNV X:g.15584534 was shown to be significantly associated with severity, being present in all moderate and severe cases, significantly different compared to the control group.

The discrepancy in SARS-CoV2 infection patterns among males and females has been extensively reviewed (44, 45). In females, *ACE 2*, X:g.15584534 was associated with the disease severity in our cohort. In a previous study, Martinez-Gomez et al. found that the T allele of rs2285666 represents a risk factor for severe COVID-19, especially for men, irrespective of age, hypertension, obesity, and diabetes (46). A limitation of our study is the gender unequal distribution in both groups (patients and controls); we have 74 (77%) males among patients and only 27 (39%) among controls. On the other hand, reports on geographically and ethnically close populations demonstrated the association of *TMPRSS2 rs12329760* and *ACE2 rs2285666* SNVs with COVID-19 disease severity (47, 48), highlighting the importance of population-specific studies. Additionally, our linkage disequilibrium findings highlighted a high correlation (D’= 0.989, R2 = 0.977) between rs879922 and rs4240157 SNVs in the *ACE2* gene in the general population which are consistent with previous findings showing segregation of both SNVs rs879922 and rs4240157 with increased severity in obese COVID-19 patients as compared to lean counterparts from the Emirati population (11). These findings extended our understanding by suggesting a potential involvement of these specific SNVs in SARS-CoV-2 infection, both within the general population and specifically among the Emirati population. However, of note, our study showed that the Emirati population lacks the previously reported *ACE2* and *TMPRSS2* variants that mark susceptibility to SARS-CoV-2 infection, similar to a study carried out on the Turkish population (49), emphasizing the significance of conducting population-specific studies upon large-scale pandemics.

SNVs were previously identified to influence cytokine levels and affect susceptibility to infections such as tuberculosis (50). In COVID-19 infection, SNVs of cytokines and chemokines were associated with the severity and clinical outcomes (51). The frequencies of rs2298659 and rs12329760 SNVs in the *TMPRSS2* gene were previously associated with the severity of COVID-19 infection (52).

Our findings about its association with various cytokines further highlight its potential in predicting disease severity. Furthermore, the correlation of *rs17854725* SNV in the *TMPRSS2* gene with inflammatory cytokines and chemokines in COVID-19 patients supports previous data, suggesting its association with disease severity (40). In addition, the association of the novel genetic variant chrX:15,584,534 in the *ACE2* gene with inflammatory cytokines in COVID-19 patients suggests its potential use in identifying individuals within the Emirati population who might be susceptible to the cytokine storms.

## Conclusion

Certain gene variants in *TMPRSS2* and *ACE2* genes are notorious for affecting the expression levels or functionality of the encoded proteins. These genetic variations can influence the interaction of the ACE2 and the virus spike protein. In addition, the cytokine levels in COVID-19 reflect the dysregulation of the immune response. Such derangement plays a significant role in the pathogenesis of the cytokine storm and can be partially linked to different disease determinants including genetic susceptibility. Interestingly, our study showed that the genetic makeup of the Emirati population lacks the previously reported *ACE2* and *TMPRSS2* variants that increase susceptibility to SARS-CoV-2 infection. In contrast, Emirati patients carry a newly reported variant of *ACE2* that increases the disease severity. Further studies are needed to decipher the link of such findings. Speculations about the potential association between *ACE2* and *TMPRSS2* gene variants and the cytokine response in COVID-19 have been made. It is hypothesized that certain genetic variations in these genes may impact the host immune response and contribute to the dysregulation of cytokine production during SARS-CoV-2 infection.

## Data availability statement

The data presented in the study are deposited in the Figshare repository, accession number: https://doi.org/10.6084/m9.figshare.24722253.v1.

## Ethics statement

The studies involving humans were approved by The Dubai Scientific Research Ethics Committee, Dubai, UAE. The studies were conducted in accordance with the local legislation and institutional requirements. The participants provided their written informed consent to participate in this study.

## Author contributions

NE: Data curation, Formal analysis, Investigation, Methodology, Validation, Visualization, Writing – original draft. AB: Data curation, Formal analysis, Investigation, Methodology, Software, Validation, Visualization, Writing – original draft. HA: Data curation, Investigation, Resources, Writing – review & editing. SA: Conceptualization, Investigation, Methodology, Validation, Visualization, Writing – original draft. SH: Investigation, Methodology, Validation, Writing – original draft. TV: Formal analysis, Investigation, Methodology, Validation, Writing – original draft. LE: Investigation, Writing – review & editing. TH: Investigation, Writing – review & editing. PG: Formal analysis, Software, Writing – original draft. NH: Methodology, Writing – original draft. HH: Investigation, Validation, Writing – review & editing. IT: Data curation, Writing – original draft, Writing – review & editing. JT: Data curation, Resources, Writing – review & editing. NS: Resources, Writing – review & editing. AM: Resources, Writing – review & editing. QH: Resources, Writing – review & editing. RH: Conceptualization, Funding acquisition, Investigation, Methodology, Resources, Supervision, Validation, Writing – review & editing. MS-A: Conceptualization, Funding acquisition, Investigation, Project administration, Resources, Writing – original draft, Writing – review & editing.
