# Supplementary material for: Association of specific ACE2 and TMPRSS2 variants with circulatory cytokines of COVID-19 Emirati patients
Source: Front Immunol. 2024 May 24;15:1348229. doi: 10.3389/fimmu.2024.1348229 (PMC11157456; doi:10.3389/fimmu.2024.1348229)
Supplement: Supplementary file 1 [file DataSheet_1.docx]

**Supplementary Tables:**

**Supplementary Table S1:** List of ACE2 and TMPRSS2 primers used for the targeted next-generation sequencing

| **Primer Name** | **Primer sequence (forward)** | **Primer sequence (reverse)** | **Gene** | **Chromosome Location** |
| --- | --- | --- | --- | --- |
| ACE2_rs191860450 | TTCATTGACTTACTTCATCTCCCAC | ATAAACTTCCTGCTCAAACAAGCAC | ACE2 | Chromosome X |
| ACE2_rs199951323 | AGCCCTCTATGGAGTCACCC | TCTTACGAGTCCCTCTGAGCA | ACE2 | Chromosome X |
| ACE2_rs150172355 | GACGAAGTAGACAAGGAATGGGT | CCCTCAAAAGGCCCTGAACC | ACE2 | Chromosome X |
| ACE2_rs140473595 | TTATTAGCACAGCTGTCCACAAACC | GATGAAACTGCACTAGTTATGCCC | ACE2 | Chromosome X |
| ACE2_rs148771870 | GTCACTGTCCCTCTTTCCCAT | CTTTTAACCTCCCAGCAAGGCT | ACE2 | Chromosome X |
| ACE2_rs147311723 | AGCCATATGGAAACAGGGGG | TGTCTAAGTGTCCCCTTTGCT | ACE2 | Chromosome X |
| ACE2_rs41303171 | AGCCATATGGAAACAGGGGG | TGTCTAAGTGTCCCCTTTGCT | ACE2 | Chromosome X |
| ACE2_rs149039346 | AATGCTTGGCACACAGGAAGA | TCTGTGCCACAAGTGAAGATGT | ACE2 | Chromosome X |
| ACE2_rs201715513 | TCAGTTCACACTGAAATGGGTT | TTCTCGTTTTCCAAAAGCCTGT | ACE2 | Chromosome X |
| ACE2_rs138390800 | TGCCTCTGTTGTCTCCCATTT | AGAATGCTGTTGTTTTGATGCTGG | ACE2 | Chromosome X |
| ACE2_rs2285666 | AAGGTTGGCAGACATCAGGT | GCATTCTTGTGGATTATCTGGG | ACE2 | Chromosome X |
| ACE2_rs73635825 | AGCCATGAGAACTCACCATGT | GCCCAACCCAAGTTCAAAGG | ACE2 | Chromosome X |
| ACE2_AX-82983125 | ACAAAGAGCCAAGTACACGAAGA | CCTGGGCTTTTCAGATTAAACCA | ACE2 | Chromosome X |
| ACE2_AX-83157332 | CCATAGCAGAGAAAGAAGCAGGT | CGACTGGAGTCCATGTGAGTA | ACE2 | Chromosome X |
| ACE2_rs4830542 | AATGGACCCACAGTCCGATG | GCTTCTAAGCAGGGCTTTGG | ACE2 | Chromosome X |
| ACE2_rs2074192 | TTAGGTTCATCAACAGCTCCATTG | TTCTTATGTGCCTCCCCAGTG | ACE2 | Chromosome X |
| ACE2_rs4240157 | GCTCAGTGAATTGGCCTCAG | AGTCTCGGCAGATCAGGATA | ACE2 | Chromosome X |
| ACE2_rs4646174 | ACCTTCAAGGCAATAAGAAGTAGGA | GCACATGTGGGCAATCTGTT | ACE2 | Chromosome X |
| ACE2_rs879922 | CAAATCCATCCTTATAACAGGTCG | TGTCTTCTTGTTACTGGCAGTTT | ACE2 | Chromosome X |
| ACE2_rs4646156 | TAGTAGAAGGGGCTGCAAATTGA | CTTTCTCCGTCTCCCCTGAAT | ACE2 | Chromosome X |
| ACE2_rs1978124 | ACCACACATACCACAATGGCA | GCCAGATGCTTTAACAAGTGCAA | ACE2 | Chromosome X |
| ACE2_rs4060 | CCCATTAGCCTTGACAGGGT | CCACCAGATCTTGCCCTCAC | ACE2 | Chromosome X |
| ACE2_rs12010448 | CCTTTTTCTTGTACACAGCATACTT | AAAGAAATGTCAAGGAAGCACTTA | ACE2 | Chromosome X |
| ACE2_rs4830983 | TCTGACAGCTCAGTACCCCA | GCACTGAGCCTACGATGATGA | ACE2 | Chromosome X |
| ACE2_rs112171234 | ATGATCGTCTGCCTTGGTGC | AGCCAGCATCCAGGTGTTAC | ACE2 | Chromosome X |
| ACE2_rs6629110 | AACGGAGAGAAGGCACTCTAC | TAACGCACTTCACTTTGCCC | ACE2 | Chromosome X |
| ACE2_rs4830974 | TCAGCTGCAAACAGCAAGTA | TTCTCCTTGTGAGGTAGCCA | ACE2 | Chromosome X |
| ACE2_ rs5936011 | ACAGTGGTGGGTGCTATGATG | GCTGAGAGCCCCTCTTTTGG | ACE2 | Chromosome X |
| ACE2_rs6632704 | CCAGAATGGATGACCAATGCTA | TGCGTCTTATTCCCATCATTGTA | ACE2 | Chromosome X |
| ACE2_rs1996225 | GTGCATTGGAACGCAGTCAA | CCAGTGTAGGGATCCCAATATTCTT | ACE2 | Chromosome X |
| ACE2_rs75979613 | ACAGGACCAATACCCAGAGA | TGGGCAAATAATCACGTGGAA | ACE2 | Chromosome X |
| ACE2_rs191860450 | TGTGTGTGTACACGCCAGTC | AGCACTCACGATTGTTGGGA | ACE2 | Chromosome X |
| ACE2_AX-83288646 | AGTTGAGCAGTGGCCTTACA | TGCCAGAAGCAAATTTAGCCAAG | ACE2 | Chromosome X |
| TMPRSS2_rs139144487 | CCCTTCCATTTGGCATAGCA | TGACTCCAAGACCAAGAACAAT | TMPRSS2 | Chromosome 14 |
| TMPRSS2_rs148125094 | ACCTGGCAAGAATCGACGTT | ACACAGAGATGCAACAGCAGA | TMPRSS2 | Chromosome 21 |
| TMPRSS2_rs150445636 | CCTGGCATACTTTTCCACGC | CACTGGCCTCCTGTTTGTCA | TMPRSS2 | Chromosome 21 |
| TMPRSS2_rs143597099 | AAGGGGGACTCCAGATGAAC | CTAAGCATGTGAGAGGCAGGT | TMPRSS2 | Chromosome 21 |
| TMPRSS2_rs2094881 | GGACGGTTGTGAGGGTAAGT | CTGGGATGTTTGACTCCCAAATG | TMPRSS2 | Chromosome 21 |
| TMPRSS2_rs112132031 | ATGTGGGACTTGTTGGGATGC | TTGGGCAGACACACTGGTTTC | TMPRSS2 | Chromosome 21 |
| TMPRSS2_rs12329760 | GTGCTGCCCCATACTCACTT | CATGGATAATCCTCCCTCTCGTG | TMPRSS2 | Chromosome 21 |
| TMPRSS2_rs140530035 | TCAAAAGGGGGACTCCAGATG | GCAGGTGACTCCGCACTATC | TMPRSS2 | Chromosome 21 |
| TMPRSS2_rs142194573 | ACGACGTCAAGGACGAAGAC | CGTTGGAGAGAAGAATGATCCCT | TMPRSS2 | Chromosome 21 |
| TMPRSS2_rs142750000 | CTTTGGCTCGAGGAATCGGA | TCTGGTGGCTGATAGGGGAT | TMPRSS2 | Chromosome 21 |
| TMPRSS2_rs61735795 | CTCACCTTTCTCCTCGGTGG | GACATAGGTGATACCCGCCTG | TMPRSS2 | Chromosome 21 |
| TMPRSS2_rs200744510 | GATTCTGCCAACCTGCTTGC | ATGGCATTGGACGGCATTTG | TMPRSS2 | Chromosome 21 |
| TMPRSS2_rs61735789 | GTGCTGCCCCATACTCACTT | CATGGATAATCCTCCCTCTCGTG | TMPRSS2 | Chromosome 21 |
| TMPRSS2_rs3787950 | CAGCAGGAAGAGAGTGGAAAGG | CAGCAGGAAGAGAGTGGAAAGG | TMPRSS2 | Chromosome 21 |
| TMPRSS2_rs61735791 | ACTGTCCCGGATGGGGATTT | TTTCAACTGTTTAGGGGTCACCA | TMPRSS2 | Chromosome 21 |
| TMPRSS2_rs564806692 | TGGTGACCCCTAAACAGTTGAA | CACCCAGCCTTGTAGTACCT | TMPRSS2 | Chromosome 21 |
| TMPRSS2_rs75603675 | CAGCACTCTCCCAGCACC | TACCAGGGTCCCGGCTC | TMPRSS2 | Chromosome 21 |
| TMPRSS2_AX-83124836 | TCCCCATGGTGTCTCCTAGT | AGGATTTGTGTTTGCTTATGCCT | TMPRSS2 | Chromosome 21 |
| TMPRSS2_AX-82888080 | TAAACACAAGGATGCCGGGG | CTTGAACTCAAGCCGCCAGA | TMPRSS2 | Chromosome 21 |
| TMPRSS2_AX-83167238 | AGAAGGGAAGAGAGACAGCCT | CTCTGGTCTGAACGTTGCACT | TMPRSS2 | Chromosome 21 |
| TMPRSS2_AX-83114430 | AGGGGGACTCCAGATGAACTT | GACAGGAGAGGGAACATCGG | TMPRSS2 | Chromosome 21 |

**Supplementary Table S2.** Summary of the identified variants in the ACE2 and TMPRSS2 genes across the studied population.

| **Variant** | **Location** | **Existing_variation** | **Consequence** | **Exon** | **Intron** | **cDNA position** | **CDS position** | **Protein_position** | **Amino acids** | **Codons** | **SIFT** | **PolyPhen** |
| --- | --- | --- | --- | --- | --- | --- | --- | --- | --- | --- | --- | --- |
| **TMPRSS2 Variants** | | | | | | | | | | | | |
| 21:g.42842771C>G | 21:42842771 | rs28524972 | intron_variant | - | 10-13 | - | - | - | - | - | - | - |
| 21:g.42845368G>A | 21:42845368 | rs145355824, COSV59828029 | synonymous_variant | 9-14 | - | 918 | 783 | 261 | S | agC/agT | - | - |
| 21:g.42845374G>A | 21:42845374 | rs2298659, COSV59827953 | synonymous_variant | 9-14 | - | 912 | 777 | 259 | G | ggC/ggT | - | - |
| 21:g.42845383A>G | 21:42845383 | rs17854725, COSV59823030 | synonymous_variant | 9-14 | - | 903 | 768 | 256 | I | atT/atC | - | - |
| 21:g.42852497C>T | 21:42852497 | rs12329760, COSV59820912 | missense_variant | 6-14 | - | 613 | 478 | 160 | V/M | Gtg/Atg | Deleterious (0.01) | probably_damaging(0.937) |
| 21:g.42861332C>T | 21:42861332 | rs429442, COSV59824149 | intron_variant | - | 4-14 | - | - | - | - | - | - | - |
| 21:g.42866422G>A | 21:42866422 | rs199824558, COSV59820898 | synonymous_variant | 3-14 | - | 234 | 99 | 33 | V | gtC/gtT | - | - |
| **ACE2 Variants** | | | | | | | | | | | | |
| X:g.15582786T>C | X:15582786 | rs1514281 | intron_variant | - | 16-17 | - | - | - | - | - | - | - |
| X:g.15582790C>T | X:15582790 | rs2074192 | intron_variant | - | 16-17 | - | - | - | - | - | - | - |
| X:g.15582966G>A | X:15582966 | rs233575, COSV53025110 | intron_variant | - | 16-17 | - | - | - | - | - | - | - |
| X:g.15584534G>A | X:15584534 | novel | intron_variant | - | 15-17 | - | - | - | - | - | - | - |
| X:g.15586964C>T | X:15586964 | rs4240157 | intron_variant | - | 14-17 | - | - | - | - | - | - | - |
| X:g.15590807C>G | X:15590807 | rs879922 | intron_variant | - | 11-17 | - | - | - | - | - | - | - |
| X:g.15597043A>T | X:15597043 | rs4646156 | intron_variant | - | 11-17 | - | - | - | - | - | - | - |

**Supplementary Table S3.** Prevalence of *ACE2* and *TMPRS22* Single Nucleotide Variants (SNVs) in entire study group.

| Gene | SNV ID | Mild COVID-19 patients | | Moderate COVID-19 patients | | Severe COVID-19 patients | | Total COVID-19 patients | | Healthy  Controls | |
| --- | --- | --- | --- | --- | --- | --- | --- | --- | --- | --- | --- |
|  |  | **N** | **%** | **N** | **%** | **N** | **%** | **N** | **%** | **N** | **%** |
| *ACE2* | **rs1514281** | 1 | 3.13 | 5 | 15.63 | 2 | 6.25 | 8 | 8.3 | 3 | 4.3 |
|  | **rs2074192** | 14 | 43.75 | 11 | 34.38 | 9 | 28.13 | 34 | 35.4 | 30 | 43.5 |
|  | **rs233575** | 22 | 68.75 | 24 | 75 | 16 | 50 | 62 | 64.6 | 45 | 65.2 |
|  | **chrX:15,584,534** | 26 | 81.25 | 32 | 100 | 32 | 100 | 90 | 93.8 | 64 | 92.8 |
|  | **rs4240157** | 20 | 62.5 | 19 | 59.38 | 16 | 50 | 55 | 57.3 | 45 | 65.2 |
|  | **rs879922** | 21 | 65.63 | 17 | 53.13 | 17 | 53.13 | 55 | 57.3 | 43 | 62.3 |
|  | **rs4646156** | 22 | 68.75 | 20 | 62.5 | 19 | 59.38 | 61 | 63.5 | 47 | 68.1 |
| *TMPRSS2* | **rs28524972** | 23 | 71.88 | 20 | 62.5 | 17 | 53.13 | 60 | 62.5 | 41 | 49.3 |
|  | **rs145355824** | 0 | 0 | 0 | 0 | 1 | 3.13 | 1 | 1.0 | 0 | 0 |
|  | **rs2298659** | 5 | 15.63 | 7 | 21.88 | 6 | 18.75 | 18 | 18.8 | 21 | 26.1 |
|  | **rs17854725** | 22 | 68.75 | 23 | 71.88 | 23 | 71.88 | 68 | 70.8 | 68 | 75.4 |
|  | **rs12329760** | 6 | 18.75 | 12 | 37.5 | 12 | 37.5 | 30 | 31.3 | 27 | 31.9 |
|  | **rs429442** | 19 | 59.38 | 15 | 46.88 | 11 | 34.38 | 45 | 46.9 | 33 | 42.0 |
|  | **rs199824558** | 2 | 6.25 | 2 | 6.25 | 0 | 0 | 4 | 4.2 | 1 | 1.4 |

**Supplementary Table S4****:** Association evaluation of *ACE2* and *TMPRSS2* SNVs with COVID-19 infection among the COVID-19 patients and healthy controls.

| Genes | SNP ID | SNVs Frequency (%)  Total Population | | X^2^-Test | OR  (95% CI) | SNVs Frequency (%)  Female | | X^2^-Test | OR  (95% CI) | SNVs Frequency (%)  Male | | X^2^-Test | OR  (95% CI) |
| --- | --- | --- | --- | --- | --- | --- | --- | --- | --- | --- | --- | --- | --- |
|  |  | **COVID-19 patients** | **Healthy Controls** |  |  | **COVID-19 patients** | **Healthy Controls** |  |  | **COVID-19 patients** | **Healthy Controls** |  |  |
| *ACE2* | **rs1514281** | 8.3 | 4.3 | 0.362 | 2  (0.51-7.83) | 18.2 | 4.8 | 0.17 | 4.4  (0.75-26.52) | 5.5 | 3.7 | 1 | 1.5  (0.16-14.1) |
|  | **rs2074192** | 35.4 | 43.5 | 0.333 | 0.7  (0.38-1.34) | 63.6 | 57.1 | 0.789 | 1.3  (0.45-3.79) | 27.4 | 22.2 | 0.798 | 1.3  (0.46-3.75) |
|  | **rs233575** | 64.6 | 65.2 | 1 | 0.9  (0.51-1.86) | 86.4 | 69.0 | 0.223 | 2.8  (0.71-11.31) | 57.5 | 59.3 | 1 | 0.9  (0.38-2.28) |
|  | **chrX:15,584,534** | 93.8 | 92.8 | 1 | 1.2  (0.34-4) | 86.4 | 90.5 | 0.684 | 0.7  (013-3.29) | 95.9 | 96.3 | 1 | 0.9  (0.89-9.02) |
|  | **rs4240157** | 57.3 | 65.2 | 0.335 | 0.7  (0.38-1.36) | 72.7 | 73.8 | 1 | 0.9  (0.29-3.03) | 52.1 | 51.9 | 1 | 1  (0.42-2.44) |
|  | **rs879922** | 57.3 | 62.3 | 0.526 | 0.8  (0.43-1.53) | 72.7 | 73.8 | 1 | 0.9  (0.29-3.02) | 53.4 | 44.4 | 0.502 | 1.4  (0.59-3.48) |
|  | **rs4646156** | 63.5 | 68.1 | 0.619 | 0.8  (0.42-1.57) | 86.4 | 73.8 | 0.346 | 2.2  (0.55-9.1) | 56.2 | 59.3 | 0.824 | 0.9  (0.36-2.16) |
| *TMPRSS2* | **rs28524972** | 62.5 | 49.3 | 0.111 | 1.7  (0.92-3.21) | 50.0 | 59.5 | 0.597 | 0.7  (0.24-1.92) | 65.8 | 33.3 | **0.006^*^** | 3.8  (1.51-9.78) |
|  | **rs145355824** | 1.0 | 0 | 1 | 1.0  (0.99-1.03) | 4.5 | 0 | 0.344 | 1.0  (0.96-1.15) | 0 | 0 | - | - |
|  | **rs2298659** | 18.8 | 26.1 | 0.339 | 0.65  (0.31-1.37) | 22.7 | 23.8 | 1 | 0.9  (0.28-3.2) | 16.4 | 29.6 | 0.165 | 0.5  (0.17-1.31) |
|  | **rs17854725** | 70.8 | 75.4 | 0.596 | 0.79  (0.39-1.60) | 72.7 | 64.3 | 0.582 | 1.5  (0.48-4.59) | 71.2 | 92.6 | **0.031^*^** | 0.2  (0.04-0.91) |
|  | **rs12329760** | 31.3 | 31.9 | 1 | 0.97  (0.49-1.89) | 31.8 | 28.6 | 1 | 1.2  (0.38-3.57) | 30.1 | 37.0 | 0.63 | 0.7  (0.29-1.85) |
|  | **rs429442** | 46.9 | 42.0 | 0.634 | 1.22  (0.65-2.27) | 54.5 | 47.6 | 0.793 | 1.3  (0.47-3.71) | 45.2 | 33.3 | 0.363 | 1.65  (0.65-4.15) |
|  | **rs199824558** | 4.2 | 1.4 | 0.401 | 2.9  (0.32-27.05) | 9.1 | 2.4 | 0.545 | 4.1  (0.35-47.95) | 2.7 | 0 | 1 | 1.0  (0.99-1.07) |

OR: Odds Ratio for the status. CI: 95% confidence interval. *Significant association (p < 0.05). Bold p-values indicate significance.

**Supplementary Table S5:** Coverage for ACE2 X:g.15584534 SNV across the different groups’ subjects.

| chrom | pos | ref | alt | DP |
| --- | --- | --- | --- | --- |
| chrX | 15584534 | G | A,<*> | 552 |
| chrX | 15584534 | G | A,<*> | 367 |
| chrX | 15584534 | G | A,<*> | 1089 |
| chrX | 15584534 | G | A,<*> | 371 |
| chrX | 15584534 | G | A,<*> | 463 |
| chrX | 15584534 | G | A,<*> | 2 |
| chrX | 15584534 | G | A,<*> | 474 |
| chrX | 15584534 | G | A,<*> | 585 |
| chrX | 15584534 | G | A,<*> | 270 |
| chrX | 15584534 | G | A,<*> | 568 |
| chrX | 15584534 | G | A,<*> | 1299 |
| chrX | 15584534 | G | A,<*> | 1470 |
| chrX | 15584534 | G | A,<*> | 387 |
| chrX | 15584534 | G | A,<*> | 768 |
| chrX | 15584534 | G | A,<*> | 605 |
| chrX | 15584534 | G | A,<*> | 465 |
| chrX | 15584534 | G | A,<*> | 437 |
| chrX | 15584534 | G | A,<*> | 490 |
| chrX | 15584534 | G | A,<*> | 534 |
| chrX | 15584534 | G | A,<*> | 1560 |
| chrX | 15584534 | G | A,<*> | 447 |
| chrX | 15584534 | G | A,<*> | 392 |
| chrX | 15584534 | G | A,<*> | 450 |
| chrX | 15584534 | G | A,<*> | 625 |
| chrX | 15584534 | G | A,<*> | 476 |
| chrX | 15584534 | G | A,<*> | 502 |
| chrX | 15584534 | G | A,<*> | 536 |
| chrX | 15584534 | G | A,<*> | 625 |
| chrX | 15584534 | G | A,<*> | 865 |
| chrX | 15584534 | G | A,<*> | 420 |
| chrX | 15584534 | G | A,<*> | 547 |
| chrX | 15584534 | G | A,<*> | 876 |
| chrX | 15584534 | G | A,<*> | 322 |
| chrX | 15584534 | G | A,<*> | 1143 |
| chrX | 15584534 | G | A,<*> | 578 |
| chrX | 15584534 | G | A,<*> | 448 |
| chrX | 15584534 | G | A,<*> | 1445 |
| chrX | 15584534 | G | A,<*> | 436 |
| chrX | 15584534 | G | A,T,<*> | 1136 |
| chrX | 15584534 | G | A,<*> | 1311 |
| chrX | 15584534 | G | A,T,<*> | 1075 |
| chrX | 15584534 | G | A,<*> | 183 |
| chrX | 15584534 | G | A,<*> | 400 |
| chrX | 15584534 | G | A,<*> | 353 |
| chrX | 15584534 | G | A,<*> | 688 |
| chrX | 15584534 | G | A,<*> | 446 |
| chrX | 15584534 | G | A,<*> | 572 |
| chrX | 15584534 | G | A,<*> | 580 |
| chrX | 15584534 | G | A,<*> | 1249 |
| chrX | 15584534 | G | A,<*> | 1279 |
| chrX | 15584534 | G | A,<*> | 853 |
| chrX | 15584534 | G | A,<*> | 470 |
| chrX | 15584534 | G | A,<*> | 627 |
| chrX | 15584534 | G | A,<*> | 1382 |
| chrX | 15584534 | G | A,<*> | 365 |
| chrX | 15584534 | G | A,<*> | 693 |
| chrX | 15584534 | G | A,<*> | 279 |
| chrX | 15584534 | G | A,<*> | 434 |
| chrX | 15584534 | G | A,<*> | 469 |
| chrX | 15584534 | G | A,<*> | 488 |
| chrX | 15584534 | G | A,<*> | 457 |
| chrX | 15584534 | G | A,<*> | 1354 |
| chrX | 15584534 | G | A,<*> | 406 |
| chrX | 15584534 | G | A,<*> | 932 |

**Supplementary Table S6:** Associations of *ACE2* variants with COVID-19 infection severity in the Emirati population using Multiple comparisons with Bonferroni correction.

| SNV ID | COVID-19 Groups | | Total cases | | | Females | | | Males | | |
| --- | --- | --- | --- | --- | --- | --- | --- | --- | --- | --- | --- |
|  |  |  | Sig | 95% CI | | Sig | 95% CI | | Sig | 95% CI | |
|  |  |  |  | LB | UB |  | LB | UB |  | LB | UB |
| rs1514281 | Mild | Moderate | .219 | -.293 | .043 | .701 | -.783 | .283 | .570 | -.242 | .072 |
|  |  | Severe | 1.000 | -.199 | .137 | .568 | -.837 | .265 | 1.000 | -.116 | .196 |
|  | Moderate | Mild | .219 | -.043 | .293 | .701 | -.283 | .783 | .570 | -.072 | .242 |
|  |  | Severe | .531 | -.074 | .262 | 1.000 | -.569 | .498 | .167 | -.032 | .282 |
|  | Severe | Mild | 1.000 | -.137 | .199 | .568 | -.265 | .837 | 1.000 | -.196 | .116 |
|  |  | Moderate | .531 | -.262 | .074 | 1.000 | -.498 | .569 | .167 | -.282 | .032 |
| rs2074192 | Mild | Moderate | 1.000 | -.200 | .387 | 1.000 | -.425 | .889 | 1.000 | -.247 | .387 |
|  |  | Severe | .593 | -.137 | .450 | .342 | -.250 | 1.107 | 1.000 | -.233 | .393 |
|  | Moderate | Mild | 1.000 | -.387 | .200 | 1.000 | -.889 | .425 | 1.000 | -.387 | .247 |
|  |  | Severe | 1.000 | -.231 | .356 | 1.000 | -.461 | .854 | 1.000 | -.307 | .327 |
|  | Severe | Mild | .593 | -.450 | .137 | .342 | -1.107 | .250 | 1.000 | -.393 | .233 |
|  |  | Moderate | 1.000 | -.356 | .231 | 1.000 | -.854 | .461 | 1.000 | -.327 | .307 |
| rs233575 | Mild | Moderate | 1.000 | -.351 | .226 | 1.000 | -.348 | .598 | 1.000 | -.452 | .236 |
|  |  | Severe | .350 | -.101 | .476 | .424 | -.203 | .774 | .759 | -.181 | .501 |
|  | Moderate | Mild | 1.000 | -.226 | .351 | 1.000 | -.598 | .348 | 1.000 | -.236 | .452 |
|  |  | Severe | .112 | -.039 | .539 | 1.000 | -.312 | .634 | .180 | -.076 | .612 |
|  | Severe | Mild | .350 | -.476 | .101 | .424 | -.774 | .203 | .759 | -.501 | .181 |
|  |  | Moderate | .112 | -.539 | .039 | 1.000 | -.634 | .312 | .180 | -.612 | .076 |
| rs4240157 | Mild | Moderate | 1.000 | -.273 | .336 | 1.000 | -.522 | .736 | 1.000 | -.338 | .375 |
|  |  | Severe | .959 | -.180 | .430 | .788 | -.364 | .935 | 1.000 | -.273 | .433 |
|  | Moderate | Mild | 1.000 | -.336 | .273 | 1.000 | -.736 | .522 | 1.000 | -.375 | .338 |
|  |  | Severe | 1.000 | -.211 | .398 | 1.000 | -.450 | .807 | 1.000 | -.295 | .418 |
|  | Severe | Mild | .959 | -.430 | .180 | .788 | -.935 | .364 | 1.000 | -.433 | .273 |
|  |  | Moderate | 1.000 | -.398 | .211 | 1.000 | -.807 | .450 | 1.000 | -.418 | .295 |
| rs879922 | Mild | Moderate | .957 | -.179 | .429 | 1.000 | -.522 | .736 | .992 | -.213 | .496 |
|  |  | Severe | .957 | -.179 | .429 | .788 | -.364 | .935 | 1.000 | -.271 | .431 |
|  | Moderate | Mild | .957 | -.429 | .179 | 1.000 | -.736 | .522 | .992 | -.496 | .213 |
|  |  | Severe | 1.000 | -.304 | .304 | 1.000 | -.450 | .807 | 1.000 | -.416 | .293 |
|  | Severe | Mild | .957 | -.429 | .179 | .788 | -.935 | .364 | 1.000 | -.431 | .271 |
|  |  | Moderate | 1.000 | -.304 | .304 | 1.000 | -.807 | .450 | 1.000 | -.293 | .416 |
| rs4646156 | Mild | Moderate | 1.000 | -.235 | .360 | 1.000 | -.348 | .598 | 1.000 | -.296 | .412 |
|  |  | Severe | 1.000 | -.203 | .391 | .424 | -.203 | .774 | 1.000 | -.310 | .390 |
|  | Moderate | Mild | 1.000 | -.360 | .235 | 1.000 | -.598 | .348 | 1.000 | -.412 | .296 |
|  |  | Severe | 1.000 | -.266 | .328 | 1.000 | -.312 | .634 | 1.000 | -.372 | .336 |
|  | Severe | Mild | 1.000 | -.391 | .203 | .424 | -.774 | .203 | 1.000 | -.390 | .310 |
|  |  | Moderate | 1.000 | -.328 | .266 | 1.000 | -.634 | .312 | 1.000 | -.336 | .372 |
| chrX:15584534 | Mild | Moderate | ***.004^*^*** | -.327 | -.048 | ***.038^*^*** | -.837 | -.020 | .098 | -.255 | .015 |
|  |  | Severe | .***004^*^*** | -.327 | -.048 | ***.045^*^*** | -.850 | -.007 | .093 | -.254 | .014 |
|  | Moderate | Mild | ***.004^*^*** | .048 | .327 | ***.038^*^*** | .020 | .837 | .098 | -.015 | .255 |
|  |  | Severe | 1.000 | -.140 | .140 | 1.000 | -.408 | .408 | 1.000 | -.135 | .135 |
|  | Severe | Mild | ***.004^*^*** | .048 | .327 | ***.045^*^*** | .007 | .850 | .093 | -.014 | .254 |
|  |  | Moderate | 1.000 | -.140 | .140 | 1.000 | -.408 | .408 | 1.000 | -.135 | .135 |

Sig: Significance; CI: Confidence Interval; LB: Lower Bound; UB: Upper Bound; *Significant difference PostHoc=Bonferroni Alpha (0.05). Bold p-values indicate significance.

**Supplementary Table S7:** Associations of *TMPRSS2* variants with COVID-19 infection severity in the Emirati population using Multiple comparisons with Bonferroni correction.

| SNV ID | COVID-19 Groups | | Total cases | | | Females | | | Males | | |
| --- | --- | --- | --- | --- | --- | --- | --- | --- | --- | --- | --- |
|  |  |  | Sig | 95% CI | | Sig | 95% CI | | Sig | 95% CI | |
|  |  |  |  | LB | UB |  | LB | UB |  | LB | UB |
| rs28524972 | Mild | Moderate | 1.000 | -.202 | .390 | 1.000 | -.521 | .914 | 1.000 | -.278 | .382 |
|  |  | Severe | .378 | -.109 | .484 | 1.000 | -.741 | .741 | .228 | -.087 | .567 |
|  | Moderate | Mild | 1.000 | -.390 | .202 | 1.000 | -.914 | .521 | 1.000 | -.382 | .278 |
|  |  | Severe | 1.000 | -.202 | .390 | 1.000 | -.914 | .521 | .499 | -.142 | .518 |
|  | Severe | Mild | .378 | -.484 | .109 | 1.000 | -.741 | .741 | .228 | -.567 | .087 |
|  |  | Moderate | 1.000 | -.390 | .202 | 1.000 | -.521 | .914 | .499 | -.518 | .142 |
| rs145355824 | Mild | Moderate | 1.000 | -.062 | .062 | 1.000 | -.289 | .289 | 1.000 | -.320 | .223 |
|  |  | Severe | .671 | -.093 | .031 | .671 | -.441 | .155 | 1.000 | -.269 | .269 |
|  | Moderate | Mild | 1.000 | -.062 | .062 | 1.000 | -.289 | .289 | 1.000 | -.223 | .320 |
|  |  | Severe | .671 | -.093 | .031 | .628 | -.431 | .146 | 1.000 | -.223 | .320 |
|  | Severe | Mild | .671 | -.031 | .093 | .671 | -.155 | .441 | 1.000 | -.269 | .269 |
|  |  | Moderate | .671 | -.031 | .093 | .628 | -.146 | .431 | 1.000 | -.320 | .223 |
| rs2298659 | Mild | Moderate | 1.000 | -.304 | .179 | 1.000 | -.714 | .499 | 1.000 | -.435 | .215 |
|  |  | Severe | 1.000 | -.272 | .210 | 1.000 | -.769 | .483 | 1.000 | -.402 | .242 |
|  | Moderate | Mild | 1.000 | -.179 | .304 | 1.000 | -.499 | .714 | 1.000 | -.215 | .435 |
|  |  | Severe | 1.000 | -.210 | .272 | 1.000 | -.642 | .571 | 1.000 | -.295 | .355 |
|  | Severe | Mild | 1.000 | -.210 | .272 | 1.000 | -.483 | .769 | 1.000 | -.242 | .402 |
|  |  | Moderate | 1.000 | -.272 | .210 | 1.000 | -.571 | .642 | 1.000 | -.355 | .295 |
| rs17854725 | Mild | Moderate | 1.000 | -.313 | .250 | 1.000 | -.404 | .868 | .578 | -.501 | .151 |
|  |  | Severe | 1.000 | -.313 | .250 | 1.000 | -.514 | .799 | .685 | -.483 | .163 |
|  | Moderate | Mild | 1.000 | -.250 | .313 | 1.000 | -.868 | .404 | .578 | -.151 | .501 |
|  |  | Severe | 1.000 | -.281 | .281 | 1.000 | -.725 | .546 | 1.000 | -.311 | .341 |
|  | Severe | Mild | 1.000 | -.250 | .313 | 1.000 | -.799 | .514 | .685 | -.163 | .483 |
|  |  | Moderate | 1.000 | -.281 | .281 | 1.000 | -.546 | .725 | 1.000 | -.341 | .311 |
| rs12329760 | Mild | Moderate | .324 | -.469 | .094 | 1.000 | -.889 | .425 | 1.000 | -.247 | .450 |
|  |  | Severe | .324 | -.469 | .094 | .849 | -.965 | .393 | .277 | -.105 | .585 |
|  | Moderate | Mild | .324 | -.094 | .469 | 1.000 | -.425 | .889 | 1.000 | -.450 | .247 |
|  |  | Severe | 1.000 | -.282 | .282 | 1.000 | -.711 | .604 | 1.000 | -.210 | .487 |
|  | Severe | Mild | .324 | -.094 | .469 | .849 | -.393 | .965 | .277 | -.585 | .105 |
|  |  | Moderate | 1.000 | -.282 | .282 | 1.000 | -.604 | .711 | 1.000 | -.487 | .210 |
| rs429442 | Mild | Moderate | .949 | -.177 | .427 | 1.000 | -.493 | .921 | 1.000 | -.117 | .114 |
|  |  | Severe | .140 | -.052 | .552 | .951 | -.444 | 1.016 | 1.000 | -.074 | .154 |
|  | Moderate | Mild | .949 | -.427 | .177 | 1.000 | -.921 | .493 | 1.000 | -.114 | .117 |
|  |  | Severe | .949 | -.177 | .427 | 1.000 | -.635 | .778 | 1.000 | -.074 | .157 |
|  | Severe | Mild | .140 | -.552 | .052 | .951 | -1.016 | .444 | 1.000 | -.154 | .074 |
|  |  | Moderate | .949 | -.427 | .177 | 1.000 | -.778 | .635 | 1.000 | -.157 | .074 |
| rs199824558 | Mild | Moderate | 1.000 | -.122 | .122 | 1.000 | -.392 | .428 | 1.000 | -.278 | .382 |
|  |  | Severe | .649 | -.060 | .185 | 1.000 | -.281 | .567 | .228 | -.087 | .567 |
|  | Moderate | Mild | 1.000 | -.122 | .122 | 1.000 | -.428 | .392 | 1.000 | -.382 | .278 |
|  |  | Severe | .649 | -.060 | .185 | 1.000 | -.285 | .535 | .499 | -.142 | .518 |
|  | Severe | Mild | .649 | -.185 | .060 | 1.000 | -.567 | .281 | .228 | -.567 | .087 |
|  |  | Moderate | .649 | -.185 | .060 | 1.000 | -.535 | .285 | .499 | -.518 | .142 |

Sig: Significance; CI: Confidence Interval; LB: Lower Bound; UB: Upper Bound; *Significant difference PostHoc=Bonferroni Alpha (0.05). Bold p-values indicate significance.

**Supplementary Table S8**: Expression Levels of different cytokines in COVID-19 patients and healthy controls

|  | **Healthy Controls**  **(Mean ± SD, pg/ml)** | **COVID-19 patients**  **(Mean ± SD, pg/ml)** |
| --- | --- | --- |
| **CD40 Ligand** | 15.14 ± 7.88 | 3982.14 **±** 2827.30 |
| **GM-CSF** | 31.23 ± 45.13 | 37.12 **±** 30.93 |
| **Granzyme B** | 5.26 ± 3.79 | 17.72 **±** 25.43 |
| **IFN-α** | 53.96 ± 60.44 | 5.09 **±** 6.18 |
| **IFN-γ** | 9.38 ± 1.75 | 19.13 **±** 33.69 |
| **IL-1α** | 3.58 ± 5.2 | 9.58 **±** 1.90 |
| **IL-1β** | 517.51 ± 1540.03 | 2.12 **±** 1.56 |
| **IL-1Ra** | 4.37 ± 3.1 | 1575.53 **±** 1842.31 |
| **IL-2** | 0.91 ± 0.58 | 2.74 **±** 1.84 |
| **IL-4** | 8.6 ± 8.97 | 1.58 **±** 1.52 |
| **IL-6** | 9.65 ± 21.62 | 297.41 **±** 1609.17 |
| **IL-8** | 62.19 ± 52.65 | 23.94 **±** 33.60 |
| **IL-10** | 23.02 ± 18.21 | 151.76 **±** 393.69 |
| **IL-12p70** | 55.62 ± 34.83 | 13.96 **±** 12.07 |
| **IL-13** | 3.08 ± 2.74 | 35.65 **±** 19.63 |
| **IL-15** | 3.65 ± 1.66 | 3.07 **±** 2.11 |
| **IL-17A** | 9.66 ± 7 | 2.95 **±** 2.13 |
| **IL-33** | 48.53 ± 29.35 | 9.91 **±** 30.36 |
| **IP-10** | 221.25 ± 118.9 | 636.70 **±** 1017.69 |
| **MCP-1** | 16.77 ± 36.87 | 463.50 **±** 586.07 |
| **MIP-1α** | 137.79 ± 53.75 | 15.48 **±** 56.53 |
| **MIP-1β** | 108.2 ± 104.97 | 151.49 **±** 198.27 |
| **PD-L1/B7-H1** | 12.45 ± 8.99 | 144.89 **±** 206.04 |
| **TNF-α** | 15.14 ± 7.88 | 12.44 **±** 12.36 |

**Supplementary Figures:**


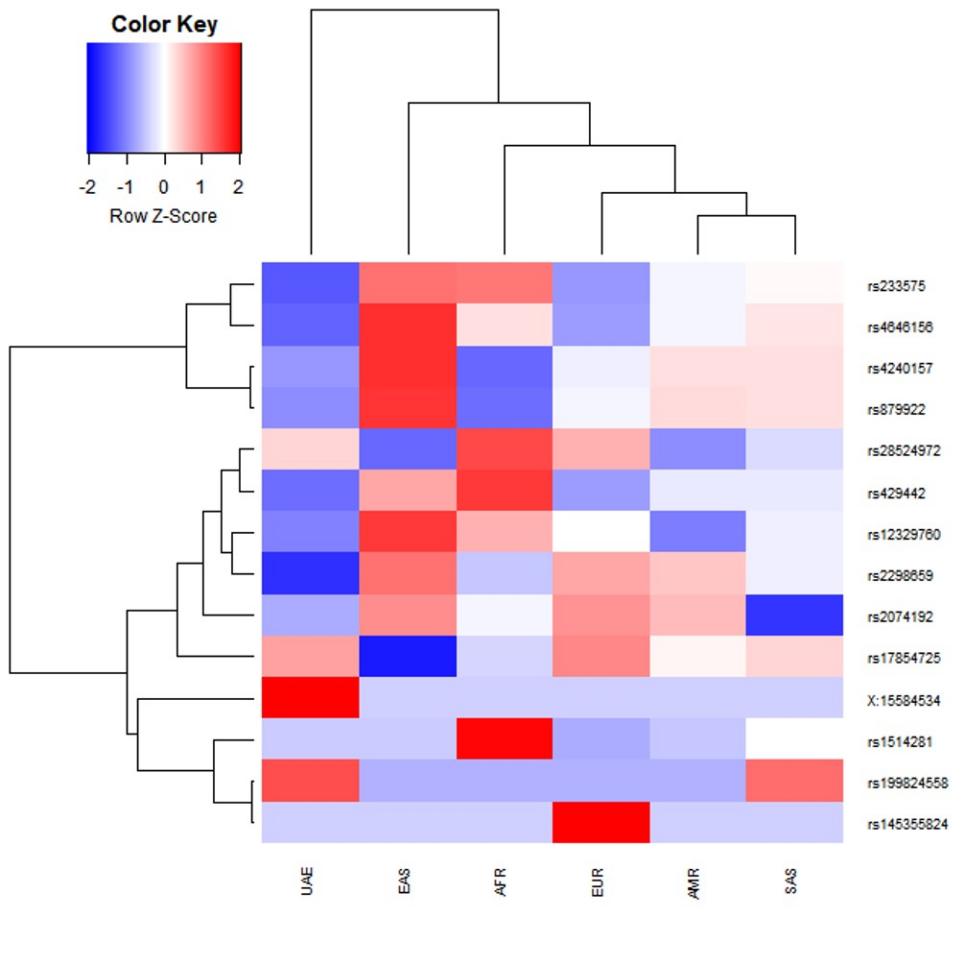


**Supplementary Figure S1.** Hierarchical clustering and heatmaps of the *ACE2* and *TMPRSS2* SNVs (rows) across (columns) different populations. AFR: African, AMR: American, EAS: East Asian, EUR: European, SAS: South Asian, UAE: Emirati.


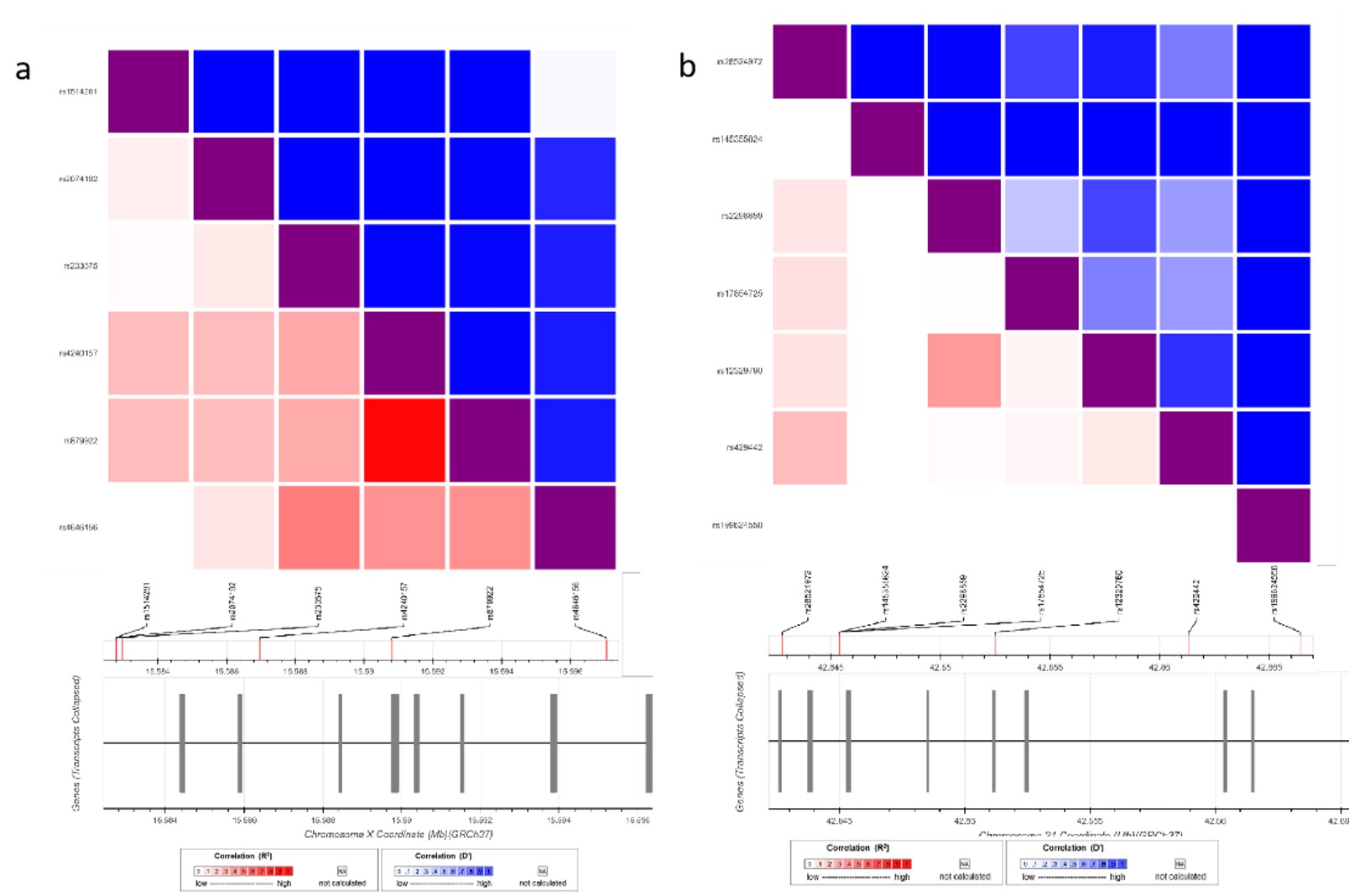


**Supplementary Figure S2.** Pairwise linkage disequilibrium statistics of a) *ACE2* and b) *TMPRSS2* variants among the general population. SNVs are presented by ordered genomic coordinates. The varying shades of blue and red indicate the strength of pairwise linkage disequilibrium based on D^’^ and R^2^ values, respectively.
